# Antimicrobial and Antibiofilm Activities of the Fungal Metabolites Isolated from the Marine Endophytes *Epicoccum nigrum* M13 and *Alternaria alternata* 13A

**DOI:** 10.3390/md19040232

**Published:** 2021-04-20

**Authors:** M. Mallique Qader, Ahmed A. Hamed, Sylvia Soldatou, Mohamed Abdelraof, Mohamed E. Elawady, Ahmed S. I. Hassane, Lassaad Belbahri, Rainer Ebel, Mostafa E. Rateb

**Affiliations:** 1 School of Computing, Engineering, & Physical Sciences, University of the West of Scotland, Paisley PA1 2BE, UK; mallique@uic.edu (M.M.Q.); Ahmedsayed.hassane@nhs.scot (A.S.I.H.); 2 National Institute of Fundamental Studies, Hantana Road, Kandy 20000, Sri Lanka; 3 National Research Centre, Microbial Chemistry Department, 33 El-Buhouth Street, Dokki, Giza 12622, Egypt; aa.shalaby@nrc.sci.eg (A.A.H.); mr.mahmoud@nrc.sci.eg (M.A.); 4 Marine Biodiscovery Centre, Department of Chemistry, University of Aberdeen, Aberdeen AB24 3UE, UK; s.soldatou@rgu.ac.uk; 5 National Research Centre, Microbial Biotechnology Department, 33 El-Buhouth Street, Dokki, Giza 12622, Egypt; ms.el-awady@nrc.sci.eg; 6 Aberdeen Royal Infirmary, Foresterhill Health Campus, Aberdeen AB25 2ZN, UK; 7 Laboratory of Soil Biology, University of Neuchatel, 2000 Neuchatel, Switzerland; lassaad.belbahri@unine.ch

**Keywords:** epicotripeptin, phragamide, *Epicoccum*, *Alternaria*, antimicrobial, antibiofilm

## Abstract

Epicotripeptin (**1**), a new cyclic tripeptide along with four known cyclic dipeptides (**2**–**5**) and one acetamide derivative (**6**) were isolated from seagrass-associated endophytic fungus *Epicoccum nigrum* M13 recovered from the Red Sea. Additionally, two new compounds, cyclodidepsipeptide phragamide A (**7**) and trioxobutanamide derivative phragamide B (**8**), together with eight known compounds (**9**–**16**), were isolated from plant-derived endophyte *Alternaria alternata* 13A collected from a saline lake of Wadi El Natrun depression in the Sahara Desert. The structures of the isolated compounds were determined based on the 1D and 2D NMR spectroscopic data, HRESIMS data, and a comparison with the reported literature. The absolute configurations of **1** and **7** were established by advanced Marfey’s and Mosher’s ester analyses. The antimicrobial screening indicated that seven of the tested compounds exhibited considerable (MIC range of 2.5–5 µg/mL) to moderate (10–20 µg/mL) antibacterial effect against the tested Gram-positive strains and moderate to weak (10–30 µg/mL) antibacterial effect against Gram-negative strains. Most of the compounds exhibited weak or no activity against the tested Gram-negative strains. On the other hand, four of the tested compounds showed considerable antibiofilm effects against biofilm forming Gram-positive and Gram-negative strains.

## 1. Introduction

Marine microorganisms are considered a rich source for drugs, drug leads, and agrochemicals [1,2,3,4]. Fungi isolated from different marine environments have been widely studied since they are cosmopolitan organisms that can survive in all phases of marine habitats regardless of environmental conditions. As a result, complex secondary metabolites are produced by marine fungi and analysis of their chemical profiles provides structurally diverse metabolites with new and novel chemical scaffolds, as well as promising biological activities [5,6].

*Alternaria* and *Epicoccum* are two ubiquitous fungal genera that are widely distributed in Nature [7,8]. They are found as endophytic, pathogenic, and saprophytic fungi [8,9]. *Alternaria* spp. are known as opportunistic pathogens that cause more than 300 plant diseases, having a detrimental impact on the agricultural economy [10]. The literature indicated that different alkaloids, terpenoids, steroids, phenolics, quinones, and pheromones are biosynthesised by more than one *Alternaria* species [11]. These metabolites are known to have potential phytotoxic, cytotoxic, antifungal and antimicrobial, and anticancer activities [11]. Meanwhile, *Epicoccum* spp. have been isolated as potential endophytic fungi residing in the sugarcane plant, which significantly impacts high root biomass and controls sugarcane pathogens [12]. Similarly, *Epicoccum* spp. are well known for their biocontrol activity against host pathogens, especially in sunflower, cotton, apple, and peaches [12]. Chemically, *Epicoccum* spp. have been shown to produce a rich number of carotenoid pigments, heterocyclic compounds, sulphur compounds, phenolics, and polysaccharides, which exhibit biologically important activities such as antioxidant, antimicrobial, herbicidal, antiviral, phytotoxic, anticancer, antitumor, and immunosuppressive [9,13,14,15,16,17]. Moreover, *E. nigrum* produces a range of diketopiperazine or cyclic dipeptides, which showed potential bioactivities such as anti-HIV, antifungal, and antibacterial [14,15]. The *Epicoccum*-derived metabolites are characterised by the presence of sulphur bridges, which possess diverse pharmacological effects. Though sulphur-containing metabolites are frequently found in Nature, the secondary metabolites with cross–ring sulphur bridges and *S*-methyl groups are rare and mainly reported from fungi [13]. 

In 2017, we initiated a collaborative project between Egypt and the UK aiming at the isolation of new endophytic fungal strains from different under-explored marine habitats in Egypt to be screened for their antimicrobial effect, with the ultimate aim of incorporating their bioactive extracts or metabolites into textiles used in Egyptian hospitals to reduce nosocomial infections. As a result, 21 out of 32 endosymbiotic marine-derived fungal isolates were isolated from both Hurghada, Red Sea and Wadi El-Natrun depression in Sahara, identified by molecular biological traits, and selected for further study based on their antimicrobial effects [18]. In this study, the chemical profiles following large scale fermentation of two endophytic fungal isolates, *Epicoccum nigrum* M13 isolated from the seagrass *Thalassia hemprichii* collected from Hurghada, Red sea and *Alternaria alternata* 13A recovered from the leaves of *Phragmites australis* collected from Wadi El-Natrun depression were studied. Herein, we report the large-scale fermentation, isolation, structure elucidation of new and known fungal metabolites of different chemical classes and their antimicrobial and antibiofilm activities on a panel of various pathogenic organisms.

## 2. Results and Discussion 

### 2.1. Isolation and Identification of the Endophytic Fungal Strains

Out of the 21 endosymbiotic marine-derived fungal isolates recovered and prioritized in our previous study, we have selected the isolates M13 and 13A for scale up due to their promising antimicrobial effects and prolific chemical profiles established by the LC-HRMS analysis. The fungal strains M13 and 13A were isolated from the marine seagrass *Thalassia hemprichii* (collected from Makady bay, south Hurghada, Egypt) and the plant *Phragmites australis* (collected from Lake El-Bida, Wadi El-Natrun, Egypt) and identified using morphological features and genetic markers (ITS rDNA and β-tubulin) as *Epicoccum nigrum* and *Alternaria alternata*, respectively, as described previously [18].

### 2.2. Fermentation, Isolation, and Structure Elucidation

**Figure 1:**
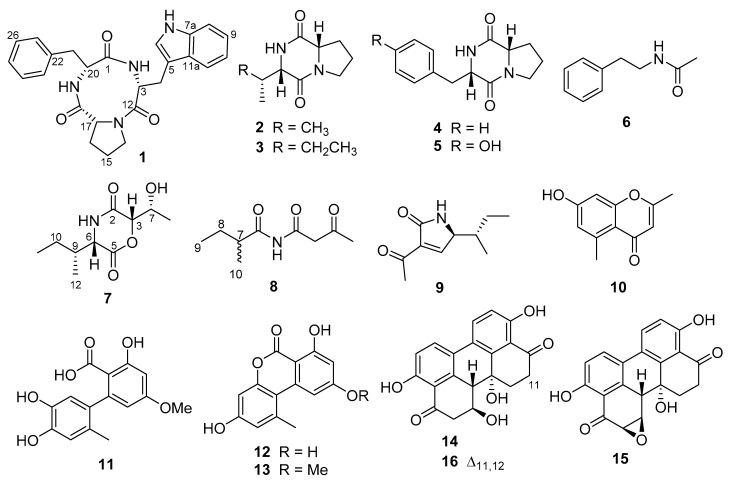
Structures of the fungal metabolites isolated from the *E. nigrum* M13 (**1**–**6**) and *A. alternata* 13A (**7**–**16**).

Large scale fermentation of the pure culture of *E. nigrum* M13 was performed on a modified marine ISP2 medium. The total extract was prepared using methanol (MeOH), defatted with *n*-hexane, and sequentially extracted with dichloromethane (DCM) and ethyl acetate (EtOAc). Both DCM and EtOAc fractions were analysed for their metabolite profiles using analytical RP–HPLC equipped with a diode array UV detector. The antimicrobial screening indicated the DCM fraction as the active subfraction, and further purification was carried out using RP-HPLC analysis which yielded six compounds (**1**–**6**, Figure 1). 

**Figure 2:**
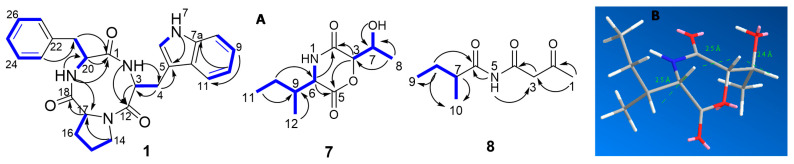
(**A**) Key COSY 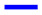 and HMBC 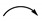 correlations of compounds **1**, **7,** and **8**; (**B**) molecular mechanics simulations of compound **7**.

Epicotripeptin (**1**) was isolated from the DCM fraction as pale yellow coloured amorphous solid. The molecular formula C_25_H_26_N_4_O_3_, indicating fifteen degrees of unsaturation, was determined by the HRESIMS analysis which showed a quasimolecular ion peak at *m/z* 453.1899 [M + Na]^+^. NMR data of compound **1** (Table 1) indicated the presence of 25 carbons which included five diastereotopic methylenes, three methines, ten aromatic/olefinic protons, four quaternary carbons, and three carbonyl carbons. The molecular formula together with the distinct ^1^H and ^13^C NMR profile indicated a peptide molecule. Analysis of 1D and 2D NMR data strongly suggested the presence of the amino acids phenylalanine (monosubstituted benzene ring at δ_H_ 7.26/H-23/27, 7.25/H-24/26, 7.19/H-25), tryptophan (1,2-disubstituted benzene ring, δ_H_ 7.36/H-8, 7.05/H-9, 6.96/H-10, 7.56/H-11, 7.17/H-6), and proline (δ_H_ 3.26–3.38/H_2_-14, 1.61–1.71/H_2_-15, 1.39–1.98/H_2_-16, 10.85, 7-NH). This assignment was corroborated by the COSY spectrum, while HMBC correlations from 7-NH to C-5, H_2_-4 to C-3 and C-5, H-3 to C-12, H-17 to C-18, H-23/25 to C-21, H_2_-21 to C-1, as well as 19-NH to C-17 and C-1 allowed for connecting the carbonyl groups and the aliphatic moieties with the respective aromatic substructures and established the sequence of the amino acids within the tripeptide (Figure 2A). 

The absolute configuration of the three amino acid residues of **1** was established using the advanced Marfey’s method [19], which upon comparison with authentic standards revealed all of them to be present as their respective L-forms. Therefore, the absolute configuration of **1** was established as cyclo(l-Trp-l-Pro-l-Phe), a new natural product for which the name epicotripeptin is proposed. 

The other isolated compounds from *E. nigrum* M13 were identified as the known cyclo(l-Pro-l-Val) (**2**) [20], cyclo(l-Pro-l-Ile) (**3**) [20], cyclo(l-Pro-l-Phe) (**4**) [21], cyclo(l-Pro-l-Tyr) (**5**) [21], and *N*- (2-phenylethyl)acetamide (**6**) [22] (Figure 1) based on their accurate mass analysis, NMR spectral, and other physical data in comparison with data reported in the literature.

Similarly, large scale fermentation of the pure culture of *A. alternata* 13A was performed on a rice medium prepared in seawater, followed by extraction and fractionation steps. Antimicrobial screening indicated that both DCM and EtOAc fractions were active, thus both were selected for further processing. Purification of the DCM and EtOAc fractions using different chromatographic techniques and finally using RP-HPLC yielded a total of 10 fungal metabolites (**7**–**16**). 

Compound **7**, obtained as pale yellow amorphous solid, had the molecular formula of C_10_H_17_NO_4_ with three degrees of unsaturation as determined by HRESIMS (*m/z* 214.1082 [M − H]^−^). The NMR data showed that the molecule contained three methyl, four methine, one diastereotopic methylene, and two carbonyl carbons (Table 2). Inspection of the NMR data revealed the presence of an isoleucine moiety in **7**, similar to the known compound tenuazonic acid (**9**), which was confirmed by the COSY spin system including H_3_-12, H_3_-11, H_2_-10, H-9, H-6, and the NH signal. Similarly, the COSY spectrum also indicated the presence of a 2-hydroxypropyl moiety, as evident from the spin system comprising δ_H_ 4.65, (H-3), δ_H_ 4.05 (H-7), δ_H_ 5.45 (OH), and δ_H_ 1.14 (H_3_-8) (Figure 2A). Two carbonyl groups at δ_C_ 168.1 (C-2) and δ_C_ 166.0 (C-5) were connected to these two substructures based on their correlations in the HMBC spectrum (Figure 2A), clearly establishing the planar structure of **7** as 3-(sec-butyl)-6-(1-hydroxyethyl)morpholine-2,5-dione, a new natural product for which the trivial name phragamide A is proposed. 

To determine the absolute configuration of the secondary alcohol at C-7, the modified Mosher ester reaction [23] was carried out. Positive Δδ were observed for the H-3, H-6, H-9, H-10, H-11, and H-12, whereas negative Δδ were observed in H-8 (Table S1), which allowed us to assign the absolute configuration as R. Based on the small coupling constant between H-3 and H-7, a syn-configuration was proposed with the chiral centre at C-3, which was thus judged to have S-configuration. The advanced Marfey’s method [19] was carried out to establish the absolute configuration of the isoleucine residue. The retention time for the FDLA-derivatized amino acid residue of **7** was compared to that of the FDLA-derivatized standard amino acid, allowing the assignment of an L-configuration (6S,9R) to the isoleucine residue. This was further confirmed by the strong ROESY correlations between H-3/H-7, H-3/H-6, and H-6/H-9, as well as the molecular mechanics simulations (Figure 2A). It is worth noting that phragamide A (**7**) is a cyclodidepsipeptide belonging to the rare dioxomorpholine class of compounds and is closely related to three fungal metabolites previously characterised from Hypericum barbatum-derived endophyte Fusarium sporotrichioides [24]. 

Compound **8** was obtained from the DCM fraction as brownish oil. HRESIMS showed a quasimolecular ion peak at *m/z* 184.0979 [M − H]^−^ indicating the molecular formula of C_9_H_15_NO_3_ with three degrees of unsaturation. ^1^H and ^13^C NMR spectra showed that the molecule contained one methine, two methylene, and three methyl groups together with three carbonyl groups and an NH proton. From the chemical shift values and the multiplicities together with the COSY spin system observed, a 2-methylbutanoyl moiety was readily identified (Table 2), similar to compound **7** (vide supra). Analysis of the remaining signals, including HMBC correlations (Figure 2A) established the planar structure of **8** as 2-methyl-*N*-(3-oxobutanoyl) butanamide, a new compound for which the trivial name phragamide B is proposed. No attempt was made to establish the absolute configuration of **8** due to the scarcity of material obtained. It is interesting to note that **8** displays a degree of chemical similarity to the well-known tenuazonic acid (**9**), which was likewise detected in the same culture of *A. alternata* 13A. 

The other isolated compounds from *A. alternata* 13A were identified as the known tenuazonic acid (**9**) [25], altechromone A (**10**) [26], altenusin (**11**) [27], alternariol (**12**) [28], alternariol monomethylether (**13**) [26], altertoxin I (**14**), altertoxin II (**15**) [29], and alterperylenol (**16**) [30] (Figure 1) based on the comparison of their accurate masses, NMR spectra, and optical rotation data with literature values.

### 2.3. Biological Activity

#### 2.3.1. Antimicrobial Activity 

The rapid development of antimicrobial resistance is considered one of the major health concerns, with pathogenic microorganisms increasingly becoming resistant to antimicrobial or anti-infective agents. If left unattended, diseases associated with drug-resistant pathogens could kill more people than cancer [31]. For the discovery of new antimicrobial agents from natural sources that could be efficient in the incorporation of textiles, we have performed initial antimicrobial screening which indicated that the total extract of each of M13 and 13A exhibited promising antimicrobial effects against a panel of pathogenic strains tested [18]. The antimicrobial activity of the pure compounds (**1**–**6**) of *E. nigrum* M13 and compounds (**7**–**16**) of *A. alternata* 13A was assessed against a panel of pathogenic microorganisms comprising Gram-positive bacteria (*Staphylococcus aureus* and *Bacillus subtilis*), Gram-negative bacteria (*Escherichia coli, Pseudomonas aeruginosa, Klebsiella pneumonia, and Proteus vulgaris*), and yeast (*Candida albicans*) (Table 3). Epicotripeptin (**1**) showed considerable activity against both Gram-positive tested bacteria, while a moderate to weak inhibition was observed against all tested Gram-negative bacteria and *C. albicans.* Cyclo(l-Pro-l-Val) (**2**) and cyclo(l-Pro-l-Ile) (**3**) displayed a weak antibacterial activity only against Gram-positive bacteria, while cyclo(l-Pro-l-Phe) (**4**) and cyclo(l-Pro-l-Tyr) (**5**) displayed a moderate antibacterial activity against Gram-positive bacteria, weak activity against Gram-negative bacteria, and no antifungal activity. 

Previous reports indicated that the diketopiperazine (DKP) derivatives isolated from different microbial sources exhibited antibacterial [32] and antifungal [33] activities. Li et al. isolated two cyclic dipeptides, cyclo(d-Pro-l-Tyr), and cyclo(l-Pro-l-Tyr) from *Lactobacillus reuteri* and found that the latter exhibited antibacterial activity against *Staphylococcus aureus* [34]. Cimmino et al. have isolated five DKPs, cyclo(l-Pro-l-Tyr), cyclo(l-Pro-l-Val), cyclo(d-Pro-d-Phe), cyclo(l-Pro-l-Leu), and cyclo(d-Pro-l-Tyr) with antibacterial activity against phytopathogenic Gram-positive bacterium *Rhodococcus fascians* LMG 3605 [35]. Cyclo(l-Pro-l-Phe), produced by *Pseudonocardia endophytica*, showed antibacterial activity against Gram-negative bacteria (*Xanthomonas campestris* and *Xanthomonas malvacearum*) and antifungal activity against (*Fusarium oxysporum* and *Fusarium solani*) [36]. Interestingly, mixing of cyclo(l-Phe-l-Pro) and cyclo(l-Leu-l-Pro) showed a good synergetic antimicrobial activity against *E. coli*, *Micrococcus luteus, S. aureus*, *C. albicans*, and *Cryptococcus neoformans* [37].

On the other hand, the MIC results for *A. alternata* 13A pure compound indicated that phragamide A (**7**) exhibited considerable antimicrobial activity against *P. aeruginosa*, *C. albicans*, and both Gram-positive strains (Table 3). Phragamide B (**8**) displayed moderate activity against *C. albicans* but showed weak activity against bacterial pathogens. Tenuazonic acid (**9**) exhibited a moderate antibacterial activity against Gram-positive strains, which is in accordance with a previous report of the antibacterial activity of **9** towards *Paenibacillus* larvae [38]. Altenusin (**11**) and alternariol (**12**) exhibited a similar antimicrobial activity towards *S. aureus, B. subtilis*, *P. aeruginosa*, and *C. albicans*. Altenusin (**11**) was reported to have antibacterial activity against *S. aureus* [39]. Moreover, Kjer et al. [40] reported strong antimicrobial activity of altenusin (**11**) against *S. aureus*, *P. aeruginosa*, and *C. albicans*. Additionally, alternariol monomethylether (**13**) and altertoxin I (**14**) showed weak antimicrobial activity against *S. aureus* and *C. albicans*, which is in agreement with previous reports by Sun et al. [25] who found that **13** had moderate antimicrobial activity against a panel of pathogenic bacteria and fungi including *S. aureus*, *Penicillium* sp., *Aspergillus* sp., and also weak antibacterial and antifungal properties observed for **12** and **13** [41]. Finally, altertoxin I (**14**), altertoxin II (**15**), and alterperylenol (**16**) exhibited a weak antibacterial activity against Gram-positive strains. Previously, **14** showed an antifungal activity against *Valsa ceratosperma*, a serious phytopathogenic fungus which causes canker disease for apples and induces the growth of lettuce seedlings [30], whereas **14** and **15** showed considerable inhibitory effect in an anti–HIV assay [29].

Our results show that out of the 16 compounds tested, only compounds **1** and **7** exhibited promising antimicrobial effects against Gram-positive strains. Moreover, all the other tested compounds showed a range of moderate, weak or no effect against the tested Gram-negative bacteria and *C. albicans*. This finding could be attributed to the loss (through isolation) of synergistic effects of the major and minor molecules present in the total fungal extract of *E. nigrum* M13 or *A. alternata* 13A which exhibited more promising results than the pure compounds alone, which is consistent with a previous study in the literature [37] and our own previous research [18]. 

#### 2.3.2. Biofilm Inhibitory Activity

**Figure 3:**
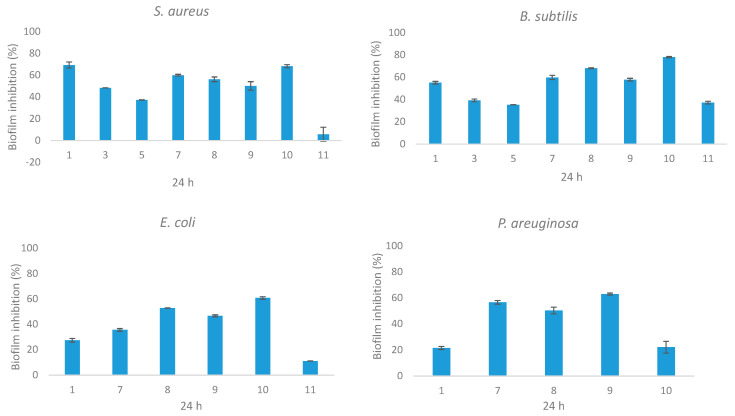
Biofilm inhibition effect of isolated pure compounds from *E. nigrum* M13 and *A. alternata* 13A against *S. aureus, B. subtilis, E. coli*, and *P. aeruginosa*. The biofilm was quantified using a microtiter plate and crystal violet assay. The bars on the graph represent the mean ± SD as a percentage of biofilm inhibition. Inactive compounds were not reported.

Bacterial biofilm formation has been found to play a critical role in the persistence of bacterial nosocomial infections. This phenomenon facilitates bacterial colonization on living or non-living surfaces and is associated with 65 to 80% of all clinical infections. Due to such adaptive changes, biofilm-forming bacteria are 10- to 1000-fold more resistant to conventional antibiotics, which thus presents a great challenge to develop antimicrobials specifically to treat biofilms [42]. Results of conventional antimicrobial susceptibility testing in vitro such as the MIC determination might not be appropriate to guide therapy for biofilm-associated infections. In fact, antimicrobial treatments based on MIC results often fail to eradicate surface-attached bacteria [43]. Consequently, we have screened the 16 pure isolated fungal metabolites against four clinical biofilm-forming pathogenic bacterial clinical isolates from Egyptian hospitals for their biofilm inhibitory activity using a microtiter biofilm plate assay. The obtained results showed that only three compounds **1**, **3**, and **5** from *E. nigrum* M13 displayed biofilm inhibitory activity against the tested clinical isolates. At 100 µg/mL, epicotripeptin (**1**) showed moderate biofilm inhibitory activity against the tested Gram-positive strains (55–70% inhibition), and weak activity against the tested Gram-negative strains (20–30%) (Figure 3). Cyclo(l-Pro-l-Ile) (**3**) and cyclo(l-Pro-l-Tyr) (**5**) exhibited a moderate biofilm formation inhibition against both Gram-positive strains but were not active against the tested Gram-negative strains. On the other hand, four pure compounds of *A. alternata* 13A were active towards the evaluated microorganisms at 100 µg/mL. Phragamides A and B (**7** and **8**), tenuazonic acid (**9**), and altechromone A (**10**) exhibited considerable biofilm formation inhibition against the tested Gram-positive strains (70–80% inhibition) and a moderate effect on Gram-negative strains (40–60%). Altenusin (**11**) exhibited moderate biofilm formation inhibition only against *B. subtilis* but a weak effect against the other three tested strains (Figure 3). 

It is worth noting that cyclic dipeptides were previously reported to exhibit biofilm inhibitory effects against Gram-positive biofilm forming strains [44,45]. Additionally, tenuazonic acid (**9**) is known to have antibiofilm activity through interference with bacterial quorum sensing [46]. It should be noted that it is difficult to interpret whether the observed effects in our study are compounded by the impact of the compound on general cell viability, as non-selective (for example, membrane lytic) activity may well be involved. Further tests using red blood cells or other mammalian cells could provide a clearer picture but were beyond the focus of the present study.

## 3. Material and Methods 

### 3.1. General Experimental Procedures

The structure characterisation of all compounds was based on ^1^H NMR, ^13^C NMR, COSY, HSQC, HMBC, and ROESY data, which were obtained on a Bruker Avance III 600 MHz spectrometer (BRUKER UK Ltd., Coventry, UK). An Agilent 1100 series HPLC system connected to the diode array G13158B detector was used for analytical and semi-preparative RP-HPLC purification (Agilent Technologies UK Ltd., Cheadle, UK). HPLC conditions were as follows: Phenomenex RP‒C18 column (Luna 5 µ, 250 × 10 mm, L × i.d.) using a gradient of MeCN in H_2_O over 35 min from 10 to 100% and ending up with 100% MeCN for 5 min at a flow rate of 1.5 mL/min. HRESIMS data were obtained using a Thermo LTQ Orbitrap coupled to an HPLC system (PDA detector, PDA autosampler, and pump). The following conditions were used: Capillary voltage of 45 V, capillary temperature of 260 °C, auxiliary gas flow rate of 10−20 arbitrary units, sheath gas flow rate of 40−50 arbitrary units, spray voltage of 4.5 kV, and mass range of 100−2000 amu (maximal resolution of 60,000). Optical rotations were recorded using a PerkinElmer 343 polarimeter (PerkinElmer, Waltham, MA, USA). UV spectra were obtained using a PerkinElmer Lambda2 UV/Vis spectrometer (PerkinElmer, Waltham, MA, USA).

### 3.2. Fungal Strains

The fungal strain M13 was obtained from *Thalassia hemprichii* leaves collected from Makady bay, South Hurghada, Red sea, Egypt and identified as *Epicoccum nigrum* based on its morphological features, together with ITS rDNA (GenBank accession number MK953943) and β-tubulin (GenBank accession number MT184348) phylogenetic analysis [18]. Strain 13A was isolated from the leaves of *Phragmites australis* collected from Lake El-Bida, Wadi El-Natrun depression, Beheira Governorate, Egypt, and identified as *Alternaria alternata* based on its morphological characteristics and its ITS rDNA sequence (GenBank accession number MK248606) [18].

### 3.3. Cultivation and Fermentation of Endophytic Fungi

The strain M13 was grown on a modified marine ISP2 broth medium (consisting of yeast extract 0.4%, malt extract 0.4%, and dextrose 0.4% for 1 L of distilled water incorporated with sea salts (consisting of 0.025% KI and MgSO_4_, 0.05% CaCl_2_ and 0.5% NaCl) adjusted to pH 6 before sterilization. Equal-sized agar plugs with mycelium were aseptically transferred to broth media (12 L) in 1 L conical flasks and incubated for 28 days under static conditions. 

For the strain 13A, a seed culture was prepared by inoculation of the pure fungal mycelia into a 250 mL Erlenmeyer flask containing 100 mL of potato dextrose-SW broth (PDB-SW) medium and incubated at 28 °C for 4 days. In addition, 5 mL of the broth culture were transferred into 1 L conical flasks (10 L) containing a rice medium: 100 g commercial rice and 100 mL 50% seawater. The flasks were incubated for 15 days at 28 °C.

### 3.4. Extraction and Isolation 

For the M13 isolate, the resulting thick mycelium bed was separated and extracted with MeOH at the end of the fermentation period. Then, about 5 g of Diaion HP20 was added to each flask containing 500 mL of broth media and shaked at 180 rpm for 5 h. Thereafter, Diaion HP20 was filtered and extracted with MeOH (3 × 500 mL). Both MeOH extracts were combined and evaporated under a vacuum to obtain the total crude extract, which was defatted with *n*‒hexane and subsequently fractionated with DCM and EtOAc. Then, DCM and EtOAc were analysed for their metabolite profile via ^1^H NMR spectroscopy and analytical HPLC (Agilent Technologies UK Ltd., Cheadle, UK, Phenomenex RP-C18 column Luna 5 μm, 250 × 4.60 mm, L × i.d.) at a flow rate of 1.0 mL/min using a gradient of 10–100% of MeCN in H_2_O over a period of 25 min and 100% MeCN for 5 min. Further purification and isolation of compounds from the DCM fraction were carried out using semi-preparative RP‒HPLC (Phenomenex RP-C18 column Luna 5 μm, 150 × 10 mm, L × i.d.) using a gradient of 10 to 100% MeCN in H_2_O over a period of 35 min followed by 100% MeCN for 5 min at a flow rate of 1.5 mL/min to produce compounds **1** (R_t_ 16.5 min, 2.0 mg), **2** (R_t_ 10.9 min, 2.1 mg), **3** (R_t_ 14.0 min, 3.6 mg), **4** (R_t_ 15.8 min, 4.5 mg), **5** (R_t_ 11.1 min, 2.0 mg), and **6** (R_t_ 18.4 min, 2.5 mg).

For the 13A isolate, after the incubation period, the fermented rice was soaked overnight in EtOAc (1:1 *v/v*), and the extract was then collected and evaporated. The obtained crude extract was suspended in 50% aqueous methanol (100 mL) and sequentially partitioned using *n*-hexane, DCM, and EtOAc. Purification of DCM and EtOAc fractions over different chromatographic techniques were carried out. Final purification of DCM extract using semi preparative HPLC over a period of 60 min yielded compounds **7** (R_t_ 14.6 min, 2.0 mg), **8** (R_t_ 25.2 min, 1.1 mg), **9** (R_t_ 19.1 min, 37 mg), **10** (R_t_ 21.6 min, 1.7 mg), **12** (R_t_ 28.9 min, 4 mg), **13** (R_t_ 38.6 min, 4.2 mg), while the EtOAc fraction over a period of 25 min following the same HPLC conditions as above furnished **11** (R_t_ 9.2 min, 2 mg), **14** (R_t_ 14.3 min, 2.0 mg), **15** (R_t_ 11.6 min, 1.4 mg), and **16** (R_t_ 18.2 min, 2.5 mg).

Epicotripeptin (**1**): Pale yellow solid; ${\left[\alpha \right]}_{D}^{25}$ = −19.65 (c 0.01, MeOH); UV (MeOH) λ_max_ (logε) 218 (3.90), 252 (3.15), 284 (2.75) nm; ^1^H NMR and ^13^C NMR data, see Table 1; HRESIMS [M + Na]^+^ at *m/z* 453.1899 C_25_H_26_N_4_O_3_Na (Calc. 453.1897).

Phragamide A (**7**): Pale yellow amorphous solid; ${\left[\alpha \right]}_{D}^{25}$ = −13.7 (*c*=0.3, MeOH), λ_max_^MeOH^: 214, 224, 254 nm; ^1^H NMR and ^13^C NMR data, see Table 2; HRESIMS: [M − H]^−^ at *m/z* 214.1082 C_10_H_17_NO_4_ (Calc. 214.1085).

Phragamide B (**8**): Brown oil; ${\left[\alpha \right]}_{D}^{25}$ = −8.3 (*c*=0.2, MeOH), λ_max_^MeOH^: 214, 224, 256 nm; ^1^H NMR and ^13^C NMR data, see Table 2; HRESIMS: [M − H]^−^ at *m/z* 184.0979 C_9_H_15_NO_3_ (Calc. 184.0979). 

### 3.5. Advanced Marfey’s Analysis 

Epicotripeptin (**1**, 0.2 mg) or phragamide A (**7**, 0.2 mg) was hydrolysed in 0.4 mL of 6 N HCl at 110 °C for 24 h. The reaction mixture was subsequently cooled, the solvent evaporated under N_2_, and residual HCl completely removed by freeze-drying the sample overnight. The hydrolysate was dissolved in 50 μL of H_2_O. To 50 μL of a 50 mM standard amino acid solution (aqueous) or the hydrolysate, 20 μL of 1 M NaHCO_3_ and 100 μL of 1% FDLA (1-fluoro-2,4-dinitrophenyl-5-l-leucinamide) in acetone were added. The reaction mixtures were incubated at 40 °C for 1 h, with frequent mixing. After the mixtures had been cooled at room temperature, the reactions were quenched by the addition of 10 μL of 2 N HCl. The samples were diluted by increasing the volume to 1 mL with MeOH. A 5 μL aliquot of each sample was analysed by LC-MS using a Kinetex C18 column (2.6 μm, 100 Å, 2.10 × 100 mm), eluted with a solvent gradient of 10 to 80% MeCN in H_2_O containing 0.1% formic acid over 30 min, at a flow rate of 1 mL/min. The comparison of the retention times of derivatised hydrolysed amino acids with that of derivatised d- and l-amino acid standards revealed the l-configuration for the amino acid residues [19].

### 3.6. Mosher Ester Analysis 

To 0.5 mg of compound **7**, 180 μL of dry pyridine-*d*_5_ was added, and the solution was transferred into an NMR tube. To initiate the reaction, 20 μL of (*R*)-MTPA-Cl was added with careful shaking and then monitored immediately by ^1^H NMR after 30 min. Analogously, for an equal amount of **7** dissolved in 180 μL of dry pyridine-*d*_5_, a second NMR tube with 20 μL of (*S*)-MTPA-Cl was reacted for 30 min, to afford the mono (*R*)-MTPA and (*S*)-MTPA ester derivatives of **7**, respectively [24]. The chemical shift difference (Δδ = δ_S-MTPA-ester_ − δ_R-MTPA-ester_) of the protons near C-7 were observed. 

### 3.7. Bioactivity Studies

#### 3.7.1. Antimicrobial Assay

For the antimicrobial testing, two Gram-positive bacteria (*Bacillus subtilis* ATCC6633 and *Staphylococcus aureus* NRRLB-767), five Gram-negative bacteria (*Escherichia coli* ATCC25955, *Pseudomonas aeruginosa* ATCC10145, *Klebsiella pneumonia* ATCCBAA-1705, and *Proteus vulgaris* ATTC7829), and one yeast (*Candida albicans* ATCC10231) were obtained from the Microbiology and Immunology Department, Faculty of Medicine, Al-Azhar University, Egypt. The antimicrobial assay and MIC were performed as described by Ingebrigtsen et al. 2016 [47], in which 10 μL of bacterial or fungal suspension at the log phase were added to 180 μL of lysogeny broth (LB), followed by the addition of 10 μL of the tested pure compounds. The final concentrations of the mixture were 50, 40, 30, 20, 10, 5, 2.5, 1.25, 0.62, 0.31, and 0.15 µg/mL. After inoculation, the plates were incubated overnight at 37 °C for 24 h. Then, the absorbance was measured at 600 nm for bacteria and 340 nm for *C. albicans* using a Spectrostar Nano Microplate Reader (BMG LABTECH GmbH, Allmendgrun, Germany). The MIC were reported as the average of the lowest concentrations with no observable growth of microorganisms. The MIC were determined in two independent experiments. Ciprofloxacin and nystatin were used as positive controls.

#### 3.7.2. Biofilm Inhibitory Activity 

The antibiofilm formation activity was performed using a microtiter plate assay. The effect of the fungal 13A and M13 crude extract and the isolated pure compounds on the biofilm formation on four biofilm-forming pathogenic bacterial clinical isolates from Egyptian hospitals *(B. subtilis, s. aureus, p. aeruginosa*, and *E. coli)* was measured in a 96-well polystyrene microtiter plate [43]. The test bacteria were first inoculated in a 100 mL Erlenmeyer flask containing LB media and incubated at 37 °C overnight in an orbital shaker at 150 rpm. Each individual well was filled with 180 μL of LB broth, then inoculated with 10 μL of pathogenic bacterial suspension and incubated for 12 h. To this, 10 µL of the test crude and pure compounds was added along with the control (without the test sample) and incubated statically at 37 °C for 24 h. After incubation, the contents of the wells were carefully removed and washed with 200 µL per well of phosphate buffer saline (PBS) pH 7.2, to remove the free-floating bacteria. The microplate was air-dried for 1 h, stained with 200 µL/well of crystal violet solution (0.1%, *w/v*), and left at room temperature for 10 min. The microplate wells were washed three times with 200 µL/well distilled water and kept for drying to remove the excess stain. To solubilise the dye, the dried microplate was washed with 200 µL/well of 95% ethanol, and the intensity was measured at an optical density of 570 nm using a Spectrostar Nano Microplate Reader (Spectrostar Nano, Belfast, Northern Ireland).

## Figures and Tables

**Table 1:** ^1^H (600 MHz) and ^13^C (150 MHz) NMR spectroscopic data for **1** (DMSO–*d*_6_, 298 K).

Position	^1^H (Mult., *J* in Hz)	^13^C, Mult.	HMBC
**Tryptophan moiety**			
1-CO		165.1, C	
2-NH	7.73 (br s)		C-3, C-4, C-12, C-17, C-18
3	4.30 (t, 5.4)	55.2, CH	C-4, C-5, C-12, C-18
4	3.24–3.07 (m)	25.8, CH_2_	C-3, C-5, C-6, C-12, C-11a
5		109.4, C	
6	7.17 (m)	124.3, CH	C-5, C-7a, C-11a
7-NH	10.85 (br s)		C-5, C-6, C-7a, C-11a
7a		136.0, C	
8	7.32 (d, 7.9)	111.2, CH	C-10, C-11a
9	7.05 (t, 7.6)	120.8, CH	C-11, C-7a
10	6.96 (t, 7.6)	118.2, CH	C-8, C-11a
11	7.56 (d, 7.8)	118.6, CH	C-5, C-9, C-7a, C-11a
11a		127.4, C	
**Proline moiety**			
12-CO		165.5, C	
14	3.38–3.26 (m)	44.5, CH_2_	C-15, C-16, C-17
15	1.71–1.61 (m)	21.8, CH_2_	C-14, C-16, C-17
16	1.98–1.39 (m)	27.7, CH_2_	C-14, C-15, C-17, C-18
17	4.08 (m)	58.4, CH	C-16, C-18
**Phenylalanine moiety**			
18-CO		168.9, C	
19-NH	7.97 (br s)		C-1, C-17, C-18, C-20, C-21
20	4.34 (t, 5.4)	55.7, CH	C-1, C-18, C-21, C-22
21	3.03 (dd, 14.6, 4.9)	35.4, CH_2_	C-1, C-20, C-22, C-23/27
22		137.3, C	
23/27	7.26 (d, 6.8)	129.8, CH	C-21, C-25
24/26	7.25 (t, 6.8)	127.9, CH	C-22
25	7.19 (m)	126.3, CH	C-23/C-27

**Table 2:** ^1^H (600 MHz) and ^13^C (150 MHz) NMR spectroscopic data for **7** and **8** (DMSO–*d*_6_, 298 K).

Position	Phragamide A (7)		Phragamide B (8)	
	δ_H_ (Mult., *J* in Hz)	δ_C_, Mult.	δ_H_ (Mult., *J* in Hz)	δ_C_, Mult.
1			2.15 (s)	30.0, CH_3_
2		168.1, C		201.9, C
3	4.65 (s)	81.8, CH	3.76 (br s)	52.9, CH_2_
4				169.1, C
5		166.0, C		
6	4.10 (br s)	57.5, CH		176.7, C
7	4.05 (m)	68.4, CH	2.50 (m)	41.8, CH
8	1.14 (d, 6.2)	18.6, CH_3_	1.51–1.33 (m)	26.3, CH_2_
9	1.93 (m)	38.3, CH	0.81 (t, 7.6)	10.9, CH_3_
10	1.41–1.28 (m)	24.5, CH_2_	0.98 (d, 6.9)	16.2, CH_3_
11	0.87 (t, 7.2)	11.8, CH_3_		
12	0.92 (d, 7.5)	14.7, CH_3_		
1-NH	8.36 (br s)			
5-NH			10.76 (br s)	
7-OH	5.45 (brs)			

**Table 3:** Minimum inhibitory concentrations (MIC) of the pure compounds (**1**–**16**) isolated from *E. nigrum* M13 and *A. alternata* 13A against bacterial and fungal pathogens.

Compound	Minimum Inhibitory Concentration (MIC, µg/mL) *						
	*S. aureus*	*B. subtilis*	*E. coli*	*K. pneumonia*	*P. vulgaris*	*P. aeruginosa*	*C. albicans*
**1**	2.5	2.5	10	20	10	30	30
**2**	50	40	-	-	-	-	-
**3**	20	20	-	-	-	-	-
**4**	10	10	30	30	30	-	-
**5**	10	10	30	30	30	-	-
**6**	40	40	-	-	-	-	40
**7**	5	5	20	-	-	10	20
**8**	30	40	30	40	40	-	50
**9**	10	10	-	-	-	-	-
**10**	-	-	-	-	-	-	-
**11**	20	20	-	-	-	30	50
**12**	20	30	-	-	-	40	50
**13**	30	30	-	-	-	-	40
**14**	30	30	-	-	-	-	40
**15**	30	20	-	-	-	-	40
**16**	30	20	-	-	-	-	30
Cip	0.62	0.31	1.25	1.25	1.25	2.5	-
Nys	-	-	-	-	-	-	5

* The average of two independent replicates, positive controls: Cip: Ciprofloxacin; Nys: Nystatin; -: not detected.

## Supplementary Materials

The following are available online at https://www.mdpi.com/article/10.3390/md19040232/s1, Table S1: Chemical shift difference of (S)–MTPA and (R)–MTPA ester of 6 (pyridine-*d*_5_ at 298 K, Figure S1–S30: 1D and 2D NMR spectra of compound **1**, **7,** and **8** and ^1^H NMR of all the known compounds.
