# Supplementary material for: Antimicrobial and Antibiofilm Activities of the Fungal Metabolites Isolated from the Marine Endophytes Epicoccum nigrum M13 and Alternaria alternata 13A
Source: Mar Drugs. 2021 Apr 20;19(4):232. doi: 10.3390/md19040232 (PMC8074750; doi:10.3390/md19040232)
Supplement: Supplementary file 1 [file marinedrugs-19-00232-s001.zip › supplementary check - MR corrected 4.23.pdf]

Supplementary Information

Antimicrobial and antibiofilm activities of the fungal metabo-lites isolated from the marine endophytes *Epicoccum nigrum* M13 and *Alternaria alternata* 13A

M. Mallique Qader [1, 2, ±], Ahmed A. Hamed [3, ±], Sylvia Soldatou [4], Mohamed Abdelraof [3], Mohamed E. El-awady [5], Ahmed S. I. Hassane [1,6], Lassaad Belbahri [7], Rainer Ebel [4,*], Mostafa E. Rateb [1,*]

1 School of Computing, Engineering, & Physical Sciences, University of the West of Scotland, Paisley PA1 2BE, Scotland, UK
2 National Institute of Fundamental Studies, Hantana Road, Kandy 20000, Sri Lanka
3 Microbial Chemistry Department, National Research Centre, 33 El-Buhouth Street, P.O. Box 12622, Dokki, Giza, Egypt
4 Marine Biodiscovery Centre, Department of Chemistry, University of Aberdeen, Aberdeen AB24 3UE, Scotland, UK
5 Microbial Biotechnology Department, National Research Centre, National Research Centre, 33 El-Buhouth Street, P.O. Box 12622, Dokki, Giza, Egypt
6 Aberdeen Royal Infirmary, Foresterhill health campus, Aberdeen AB25 2ZN, Scotland, UK
7 Laboratory of Soil Biology, University of Neuchatel, 2000 Neuchatel, Switzerland

* Correspondence: Rainer Ebel and Mostafa E. Rateb

Table

Table S1: Chemical shift difference of (*S*)–MTPA and (*R*)–MTPA ester of **6** (pyridine-d_5 at 298 K)

Figures

Figure S1: ^{1}H NMR spectrum of compound **1** (600 MHz, DMSO– d_6, 298 K)
Figure S2: ^{13}C NMR spectrum of compound **1** (150 MHz, DMSO– d_6, 298 K)
Figure S3: ^{1}H-^{1}H COSY spectrum of compound **1**
Figure S4: HSQC spectrum of compound **1**
Figure S5: HMBC spectrum of compound **1**
Figure S6: ^{1}H NMR spectrum of compound **2** (600 MHz, DMSO– d_6, 298 K)
Figure S7: ^{1}H NMR spectrum of compound **3** (600 MHz, DMSO– d_6, 298 K)
Figure S8: ^{1}H NMR spectrum of compound **4** (600 MHz, DMSO– d_6, 298 K)
Figure S9: ^{1}H NMR spectrum of compound **5** (600 MHz, DMSO– d_6, 298 K)
Figure S10: ^{1}H NMR spectrum of compound **6** (600 MHz, DMSO– d_6, 298 K)
Figure S11: HRESIMS spectrum of compound **7**
Figure S12: ^{1}H NMR spectrum of compound **7** (600 MHz, DMSO– d_6, 298 K)
Figure S13: ^{13}C NMR spectrum of compound **7** (600 MHz, DMSO– d_6, 298 K)
Figure S14: HSQC spectrum of compound **7**
Figure S15: ^{1}H-^{1}H COSY spectrum of compound **7**
Figure S16: HMBC spectrum of compound **7**
Figure S17: HRESIMS spectrum of compound **8**

Figure S18: ^{1}H NMR spectrum of compound **8** (600 MHz, DMSO– *d*$_6$, 298 K)
Figure S19: ^{13}C NMR spectrum of compound **8** (600 MHz, DMSO– *d*$_6$, 298 K)
Figure S20: HSQC spectrum of compound **8**
Figure S21: ^{1}H-^{1}H COSY spectrum of compound **8**
Figure S22: HMBC spectrum of compound **8**
Figure S23: ^{1}H NMR spectrum of compound **9** (600 MHz, DMSO– *d*$_6$, 298 K)
Figure S24: ^{1}H NMR spectrum of compound **10** (600 MHz, DMSO– *d*$_6$, 298 K)
Figure S25: ^{1}H NMR spectrum of compound **11** (600 MHz, DMSO– *d*$_6$, 298 K)
Figure S26: ^{1}H NMR spectrum of compound **12** (600 MHz, DMSO– *d*$_6$, 298 K)
Figure S27: 1H NMR spectrum of compound **13** (600 MHz, DMSO– d6, 298 K)
Figure S28: ^{1}H NMR spectrum of compound **14** (600 MHz, DMSO– *d*$_6$, 298 K)
Figure S29: ^{1}H NMR spectrum of compound **15** (600 MHz, DMSO– *d*$_6$, 298 K)
Figure S30: ^{1}H NMR spectrum of compound **16** (600 MHz, DMSO– *d*$_6$, 298 K)

Table

Table S1. Chemical shift difference of (*S*)–MTPA and (*R*)–MTPA ester of **6** (pyridine-*d*$_5$ at 298 K)

			$\Delta\delta = (\delta_{S\text{-ester}} - \delta_{R\text{-Ester}})$	
**C/H No**	$\delta_{S\text{-Ester}}$ **(ppm)**	$\delta_{R\text{-Ester}}$ **(ppm)**	**ppm**	**Hz (500 MHz)**
**3**	4.79	4.78	+0.01	+5
**6**	4.68	4.67	+0.01	+5
**9**	2.31	2.30	+0.01	+5
**10**	0.86	0.85	+0.01	+5
**11**	1.27, 1.48	1.25, 1.48	+0.02	+10
**12**	1.13	1.12	+0.01	+5
**8**	1.66	1.67	–0.01	–5

Figures

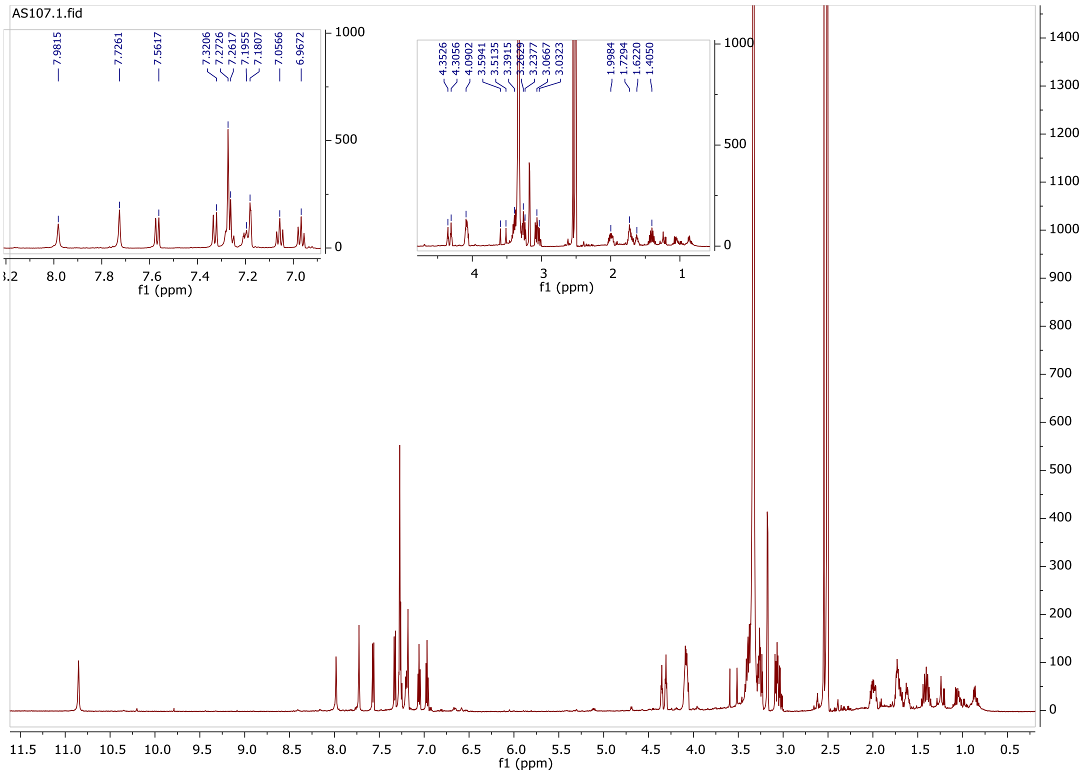

Figure S1: ^{1}H NMR spectrum of compound **1** (600 MHz, DMSO– *d*6, 298 K)

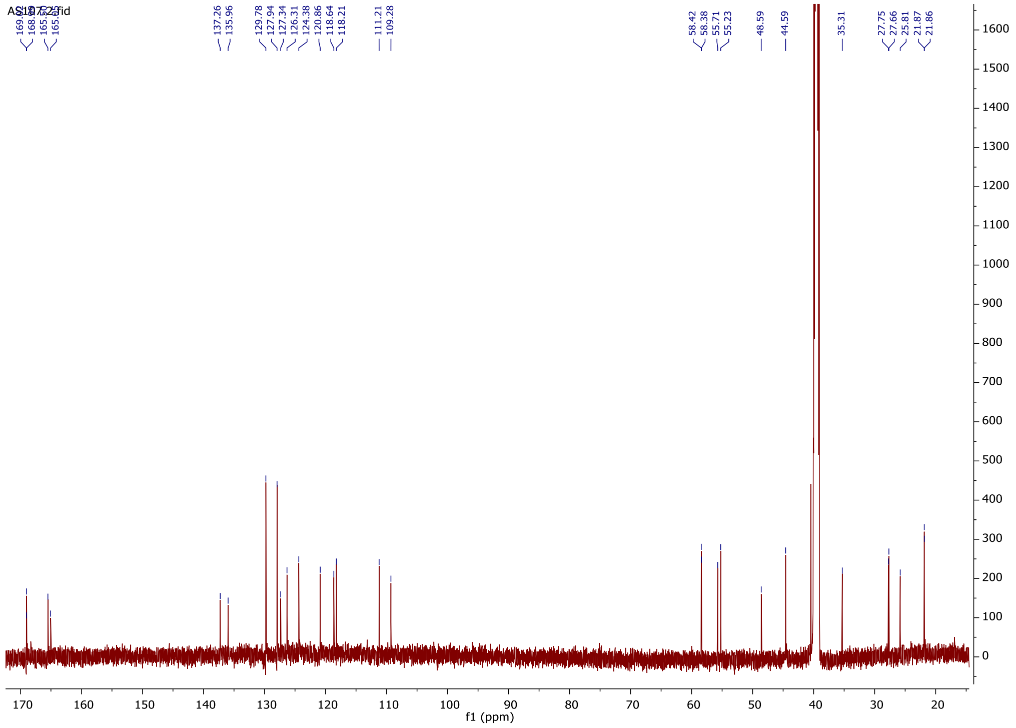

Figure S2: ^{13}C NMR spectrum of compound **1** (150 MHz, DMSO– *d*6, 298 K)

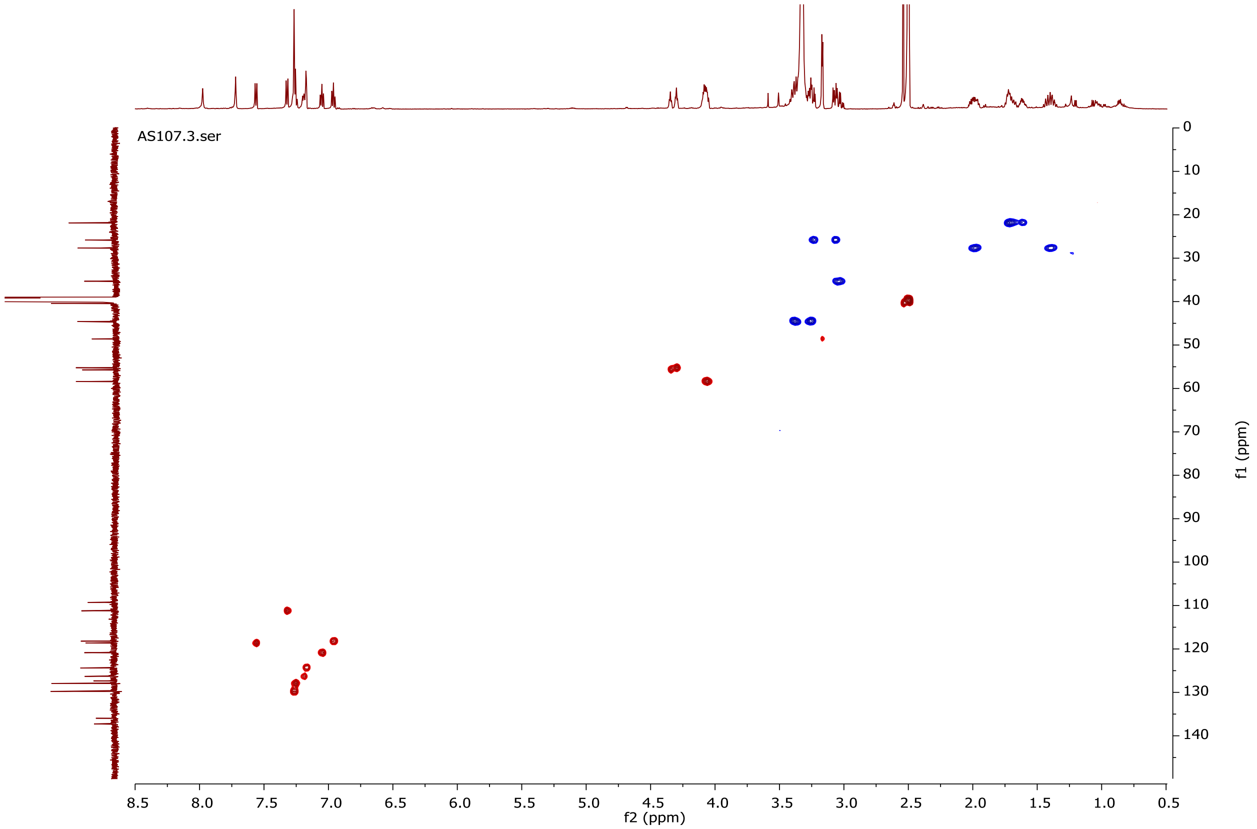

Figure S3: HSQC spectrum of compound **1** (DMSO– d_6, 298 K)

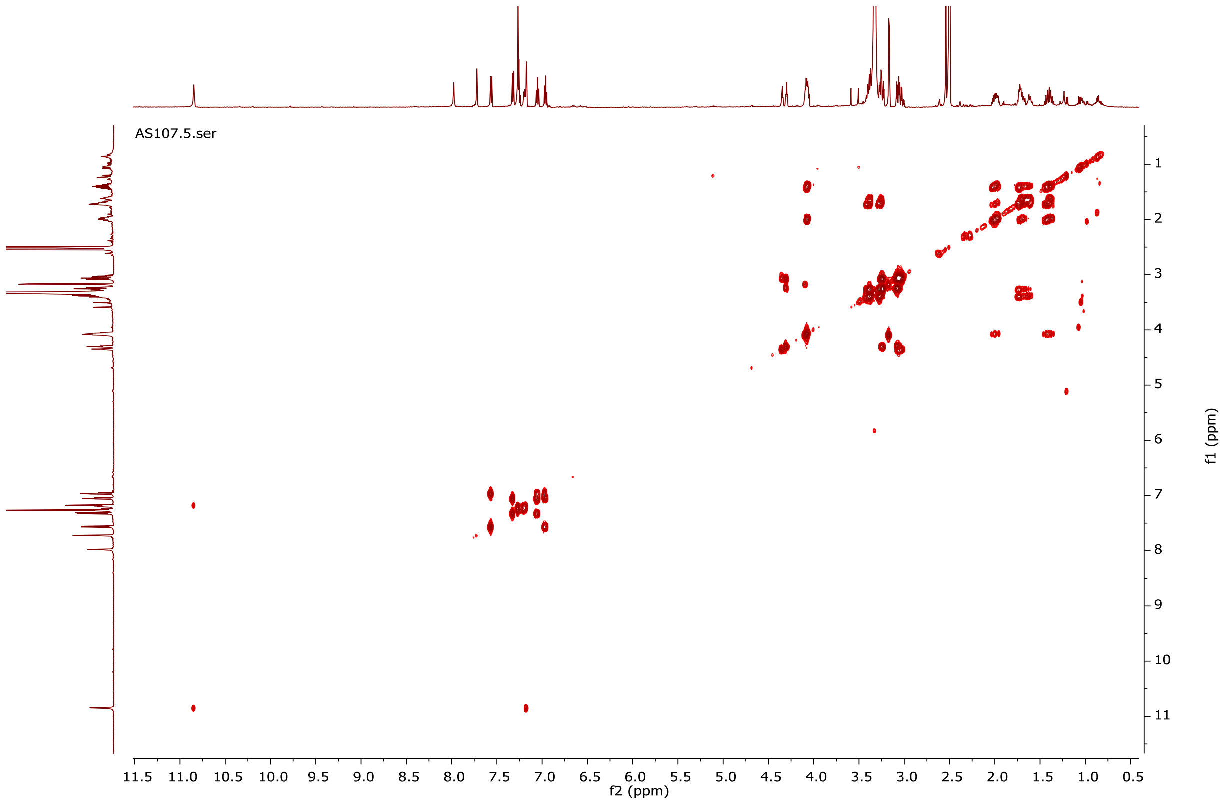

Figure S4: ^1H-^1H COSY spectrum of compound **1** (DMSO– d_6, 298 K)

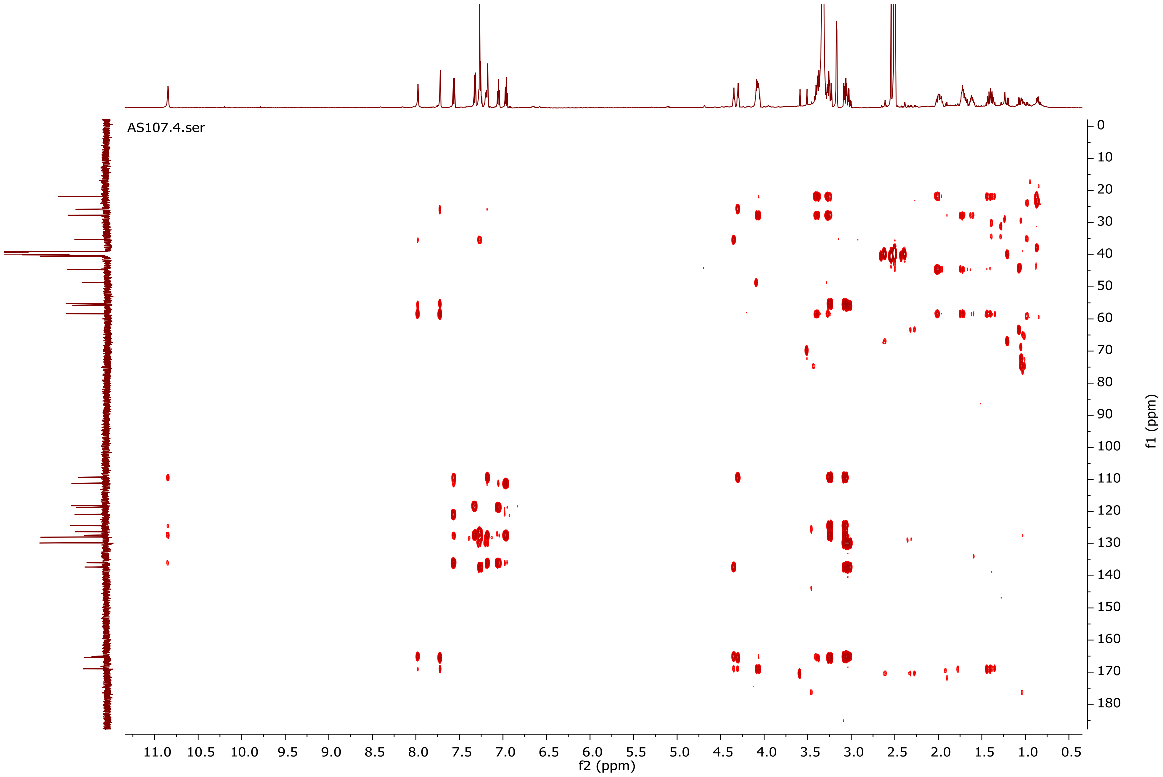

Figure S5: HMBC spectrum of compound **1** (DMSO–*d*6, 298 K)

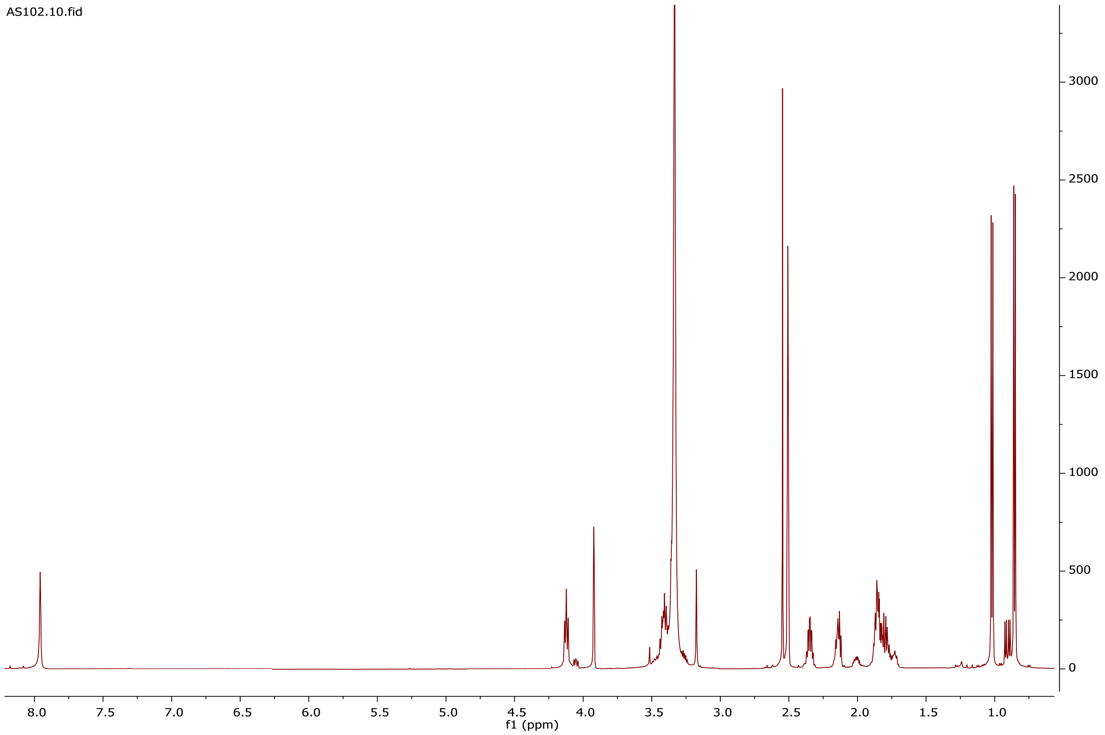

Figure S6: 1H NMR spectrum of compound **2** (600 MHz, DMSO– *d*6, 298 K)

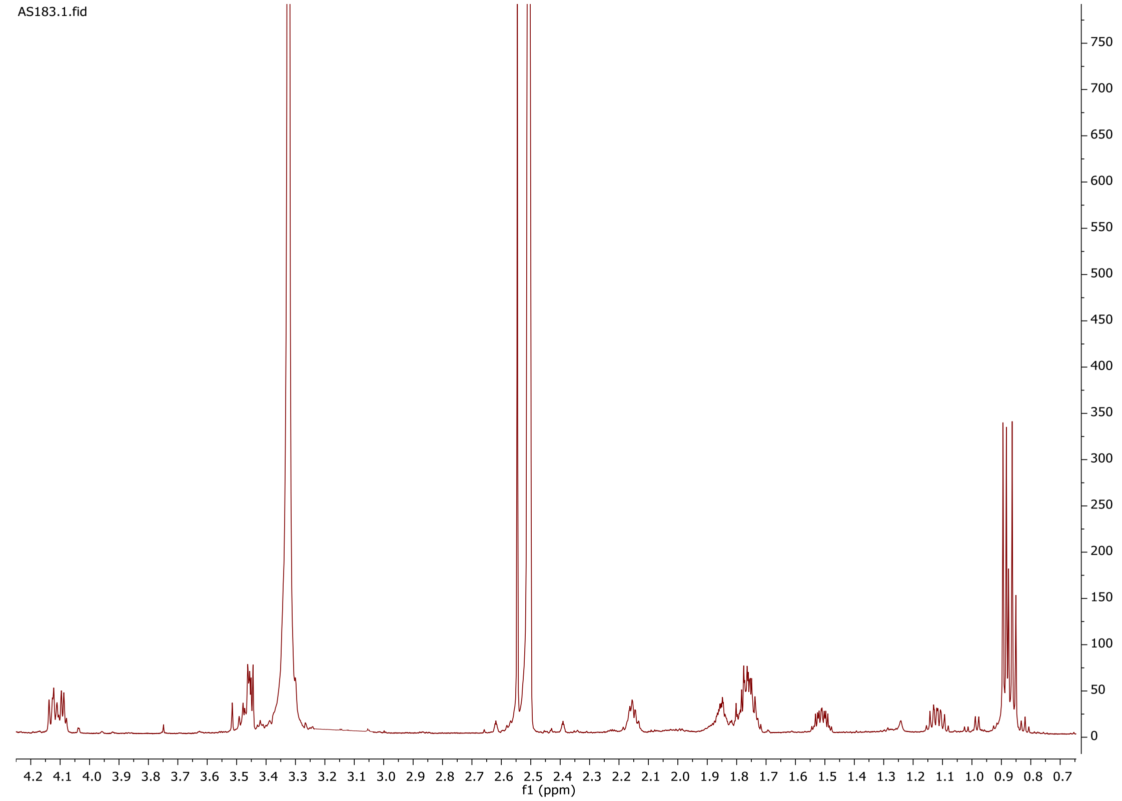

Figure S7: ^{1}H NMR spectrum of compound **3** (600 MHz, DMSO– d_6, 298 K)

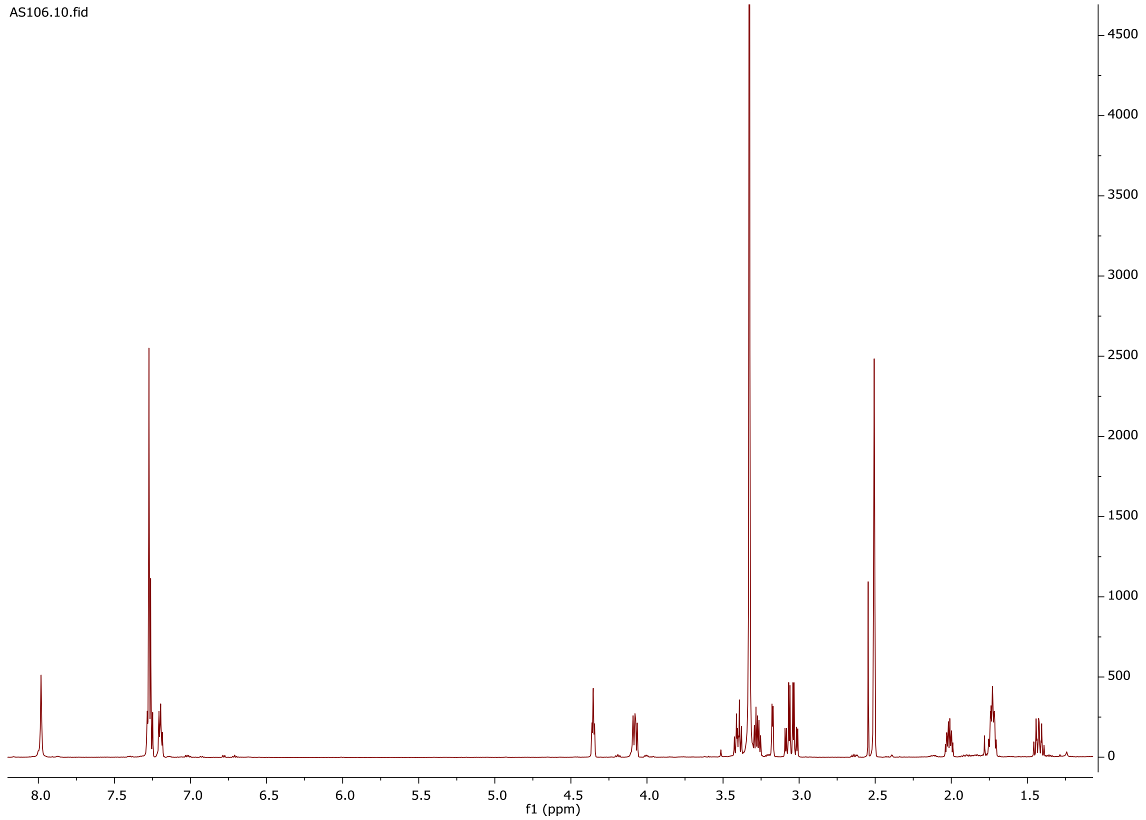

Figure S8: ^{1}H NMR spectrum of compound **4** (600 MHz, DMSO– d_6, 298 K)

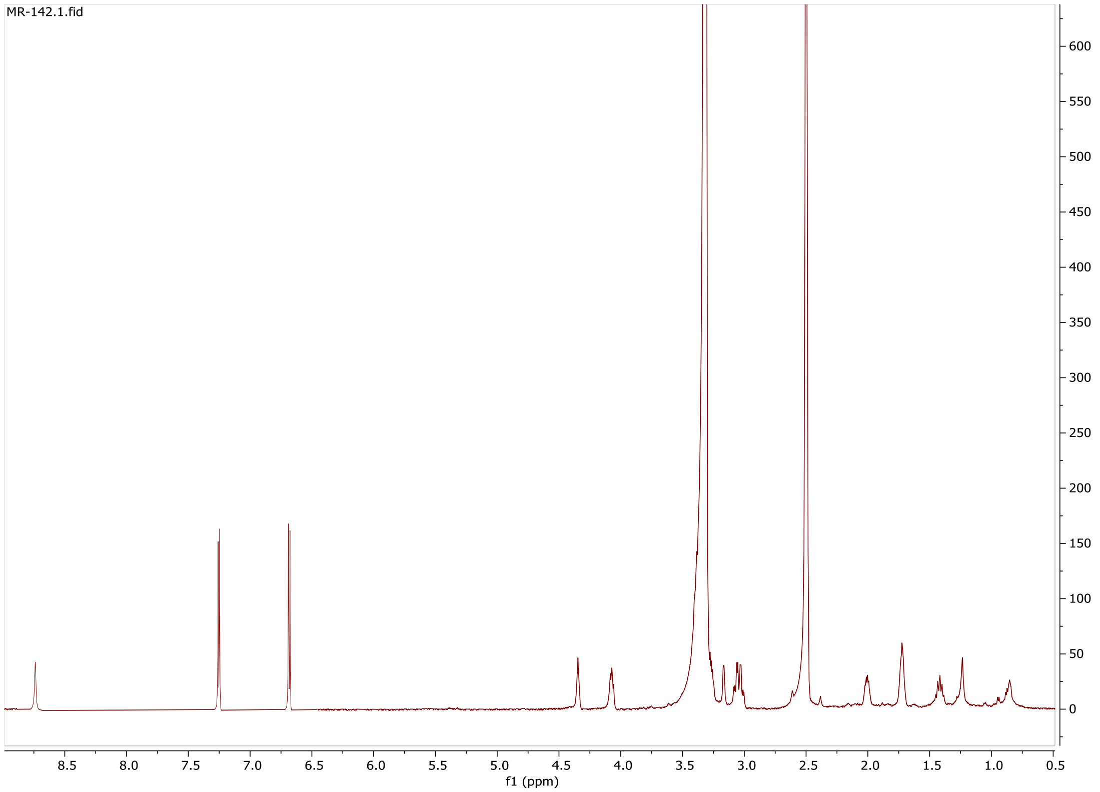

Figure S9: ^{1}H NMR spectrum of compound **5** (600 MHz, DMSO– d_6, 298 K)

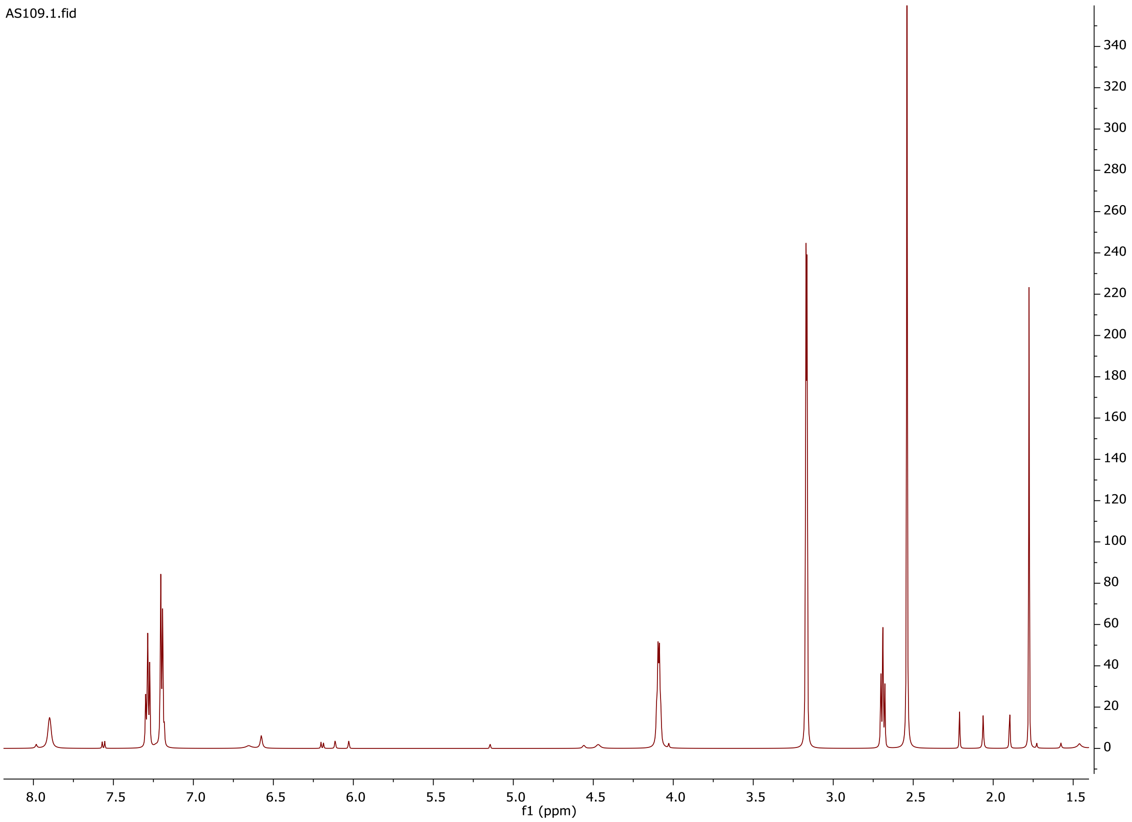

Figure S10: ^{1}H NMR spectrum of compound **6** (600 MHz, DMSO– d_6, 298 K)

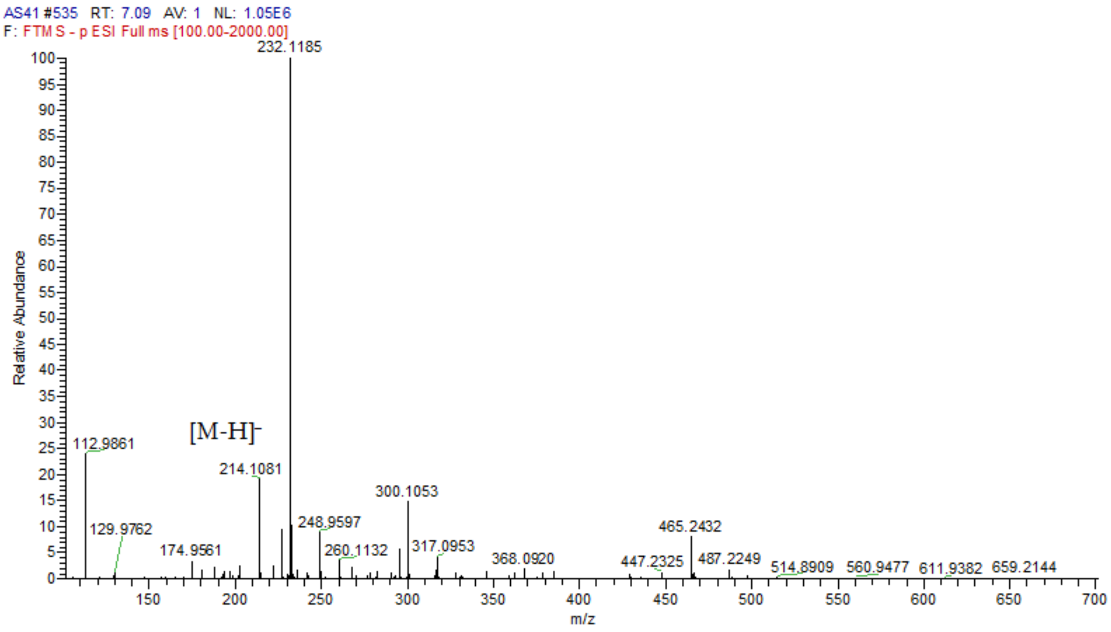

Figure S11: HRESIMS spectrum of compound **7**

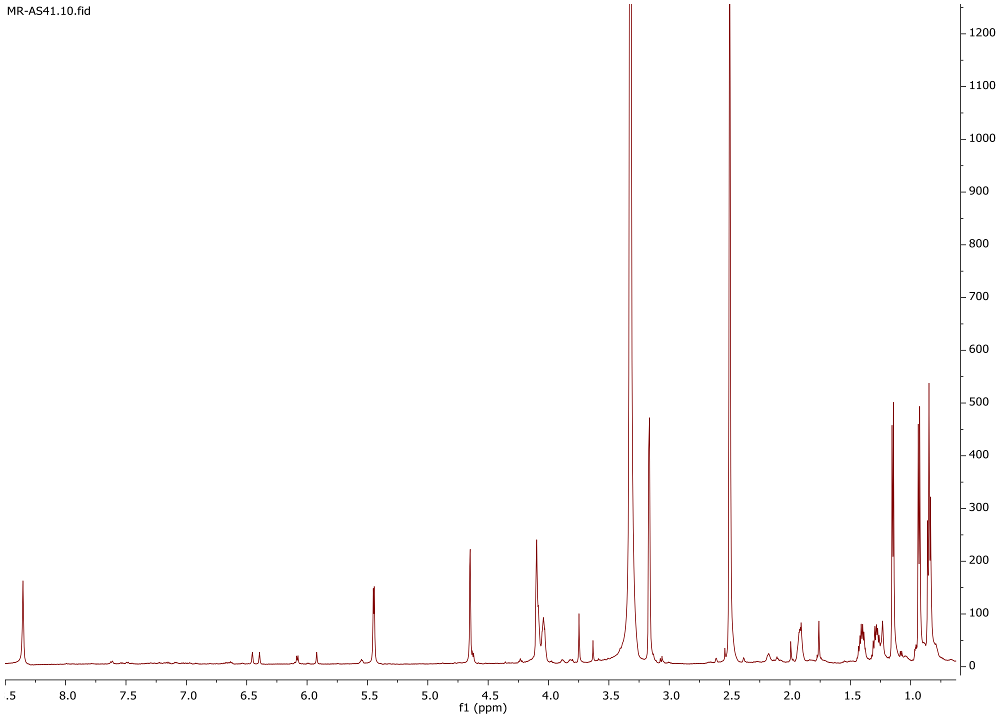

Figure S12: ^{1}H NMR spectrum of compound **7** (600 MHz, DMSO– d_6, 298 K)

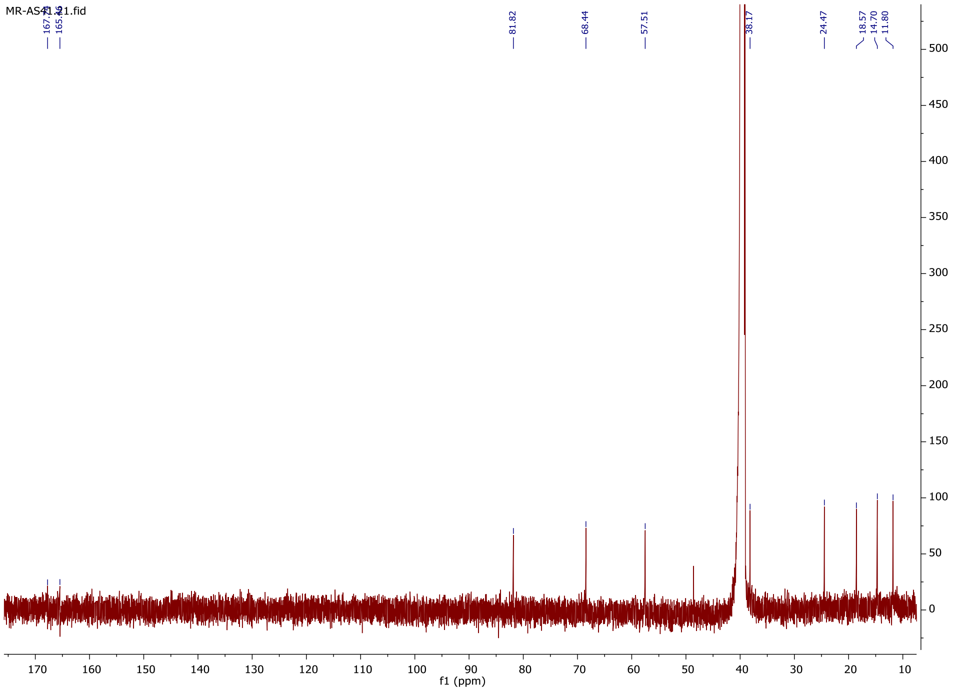

Figure S13: ^{13}C NMR spectrum of compound **7** (600 MHz, DMSO– d_6, 298 K)

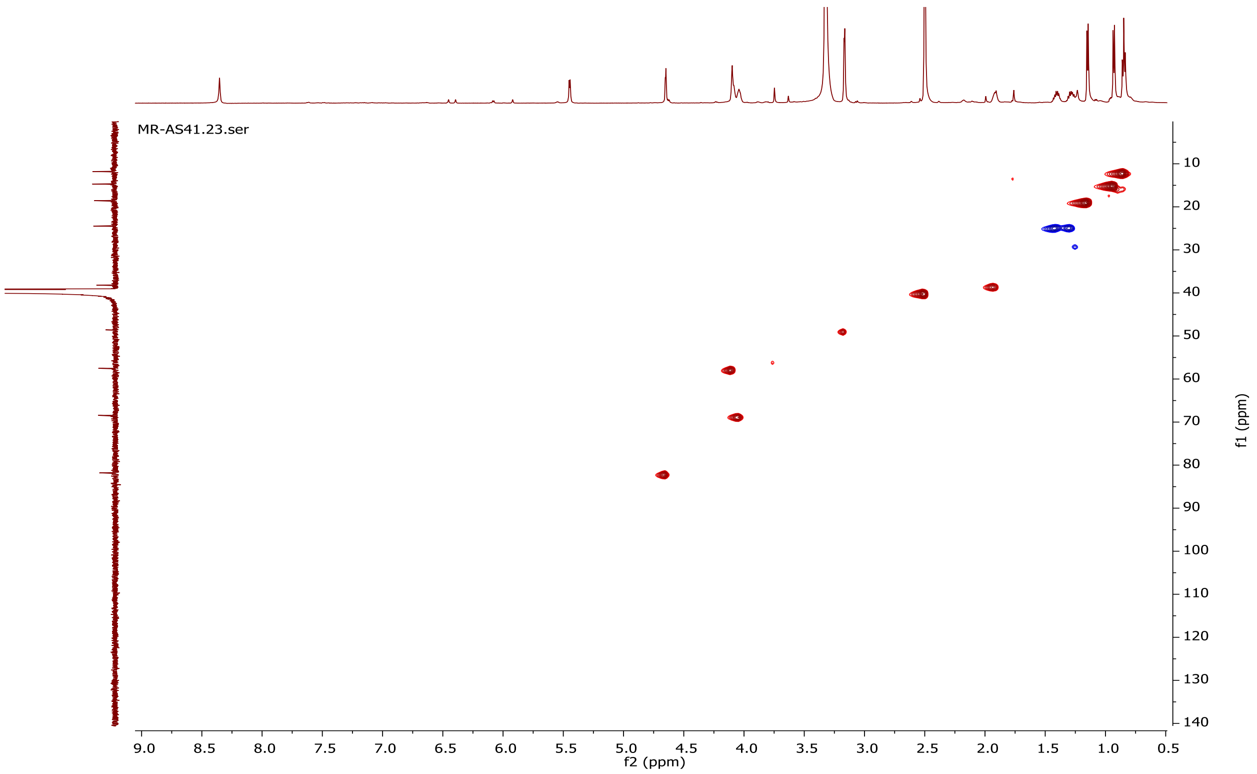

Figure S14: HSQC spectrum of compound **7** (DMSO– d_6, 298 K)

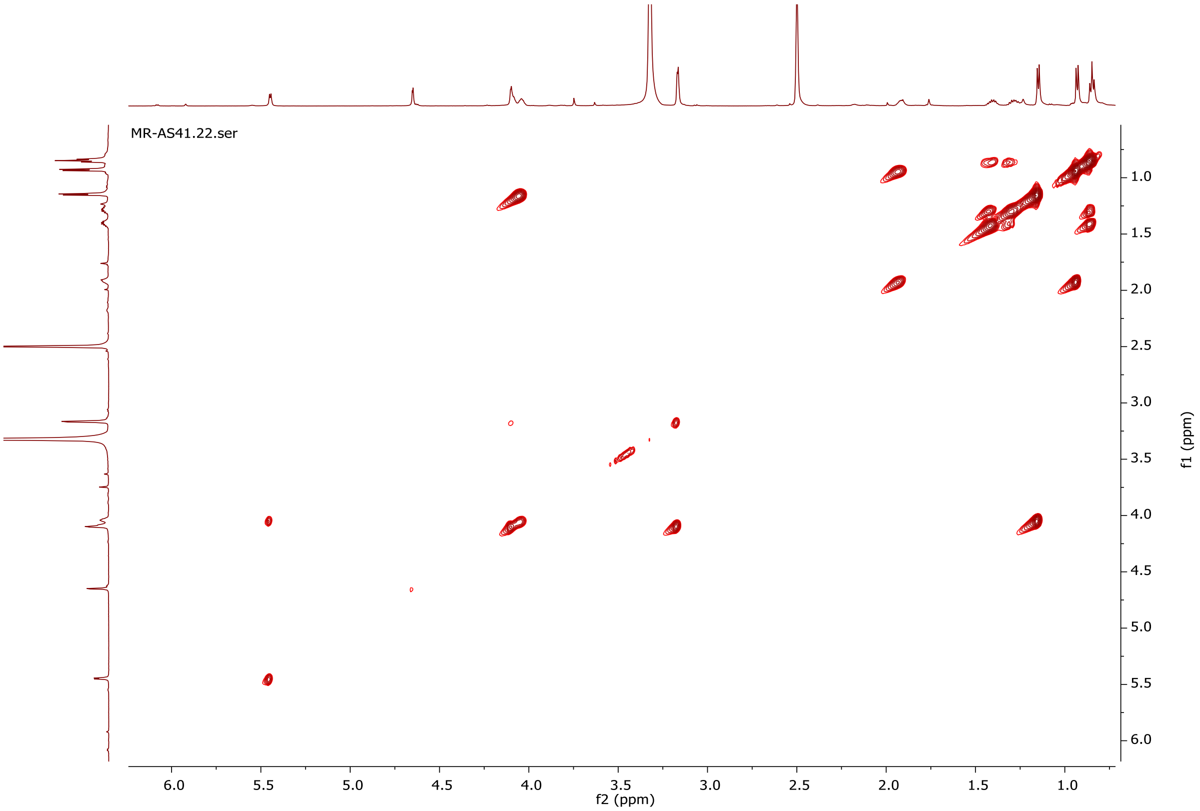

Figure S15: ${}^{1}H$-${}^{1}H$ COSY spectrum of compound **7** (DMSO– *d*$_6$, 298 K)

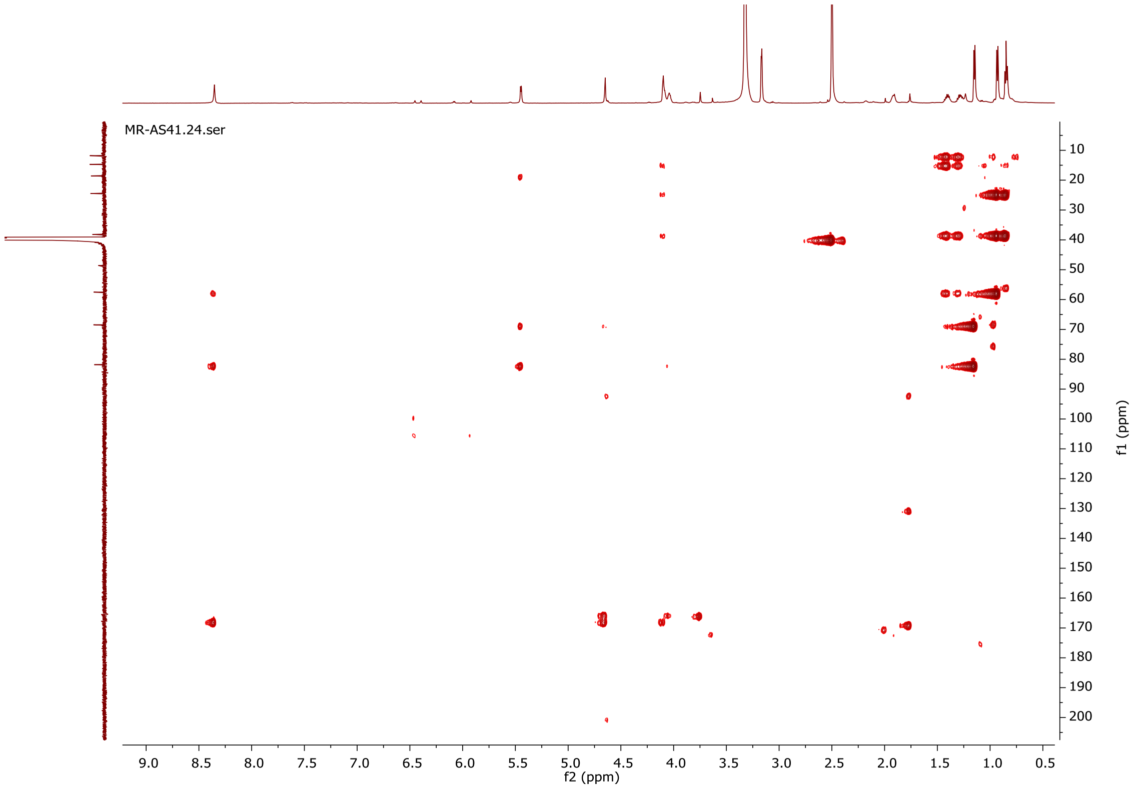

Figure S16: HMBC spectrum of compound **7** (DMSO– *d*$_6$, 298 K)

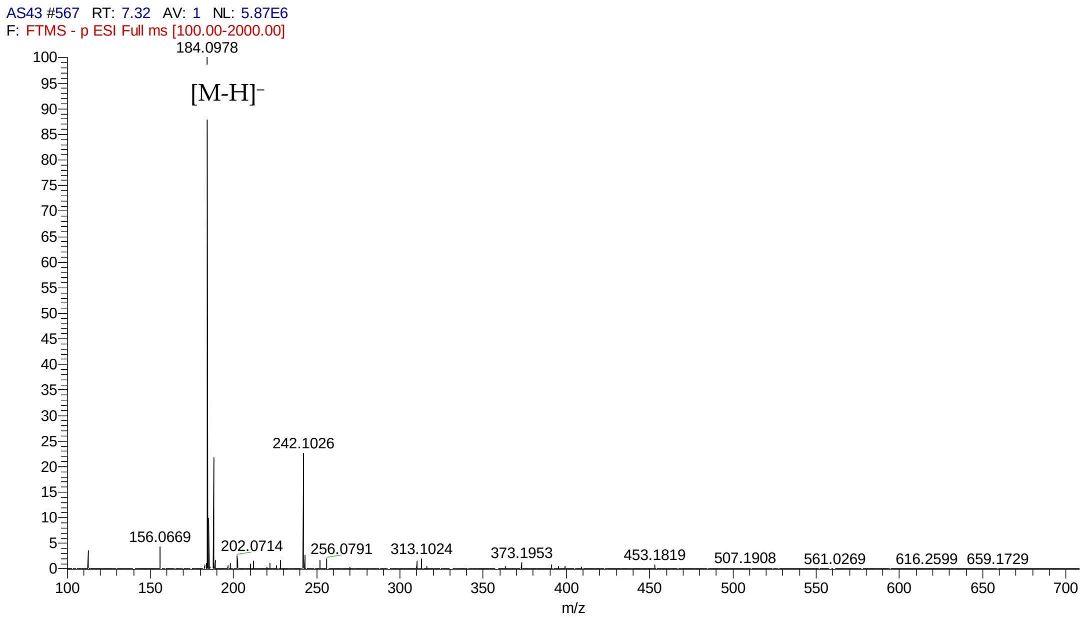

Figure S17: HRESIMS spectrum of compound **8**

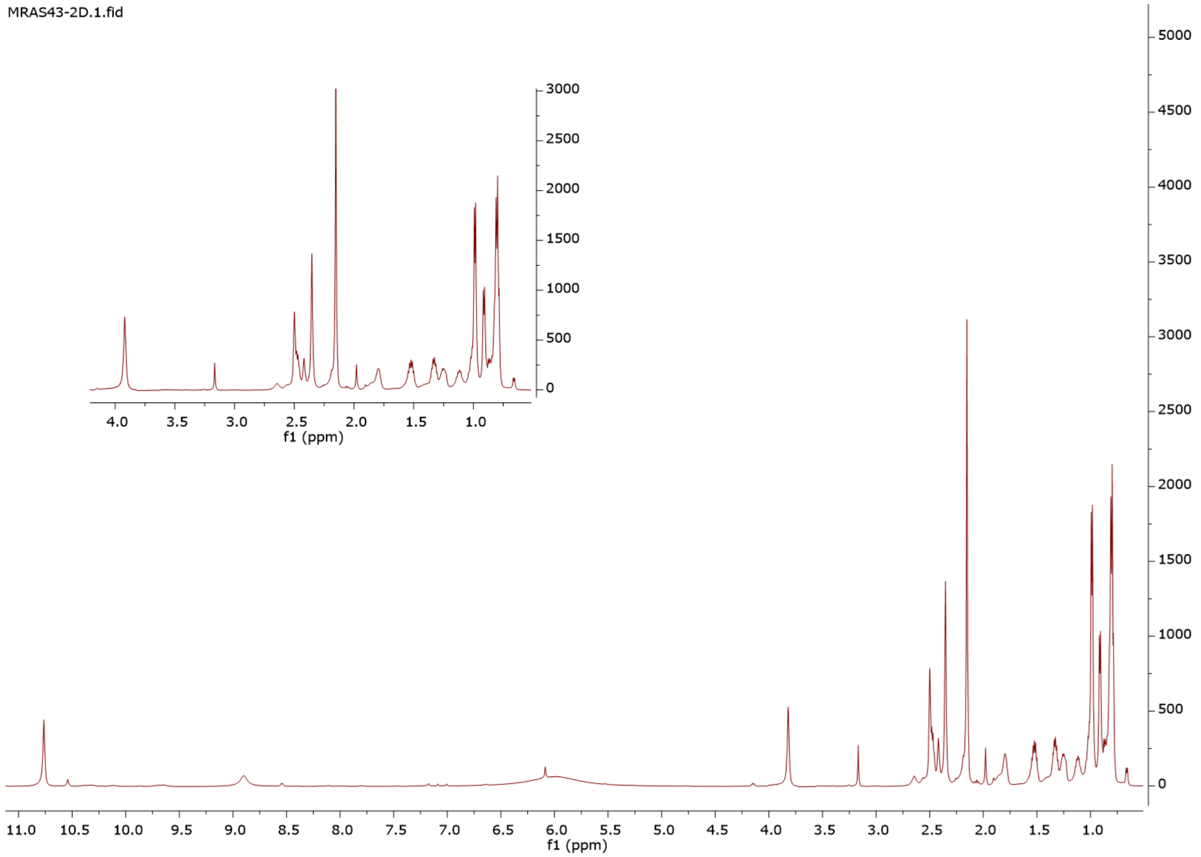

Figure S18: ^{1}H NMR spectrum of compound **8** (600 MHz, DMSO– d_6, 298 K)

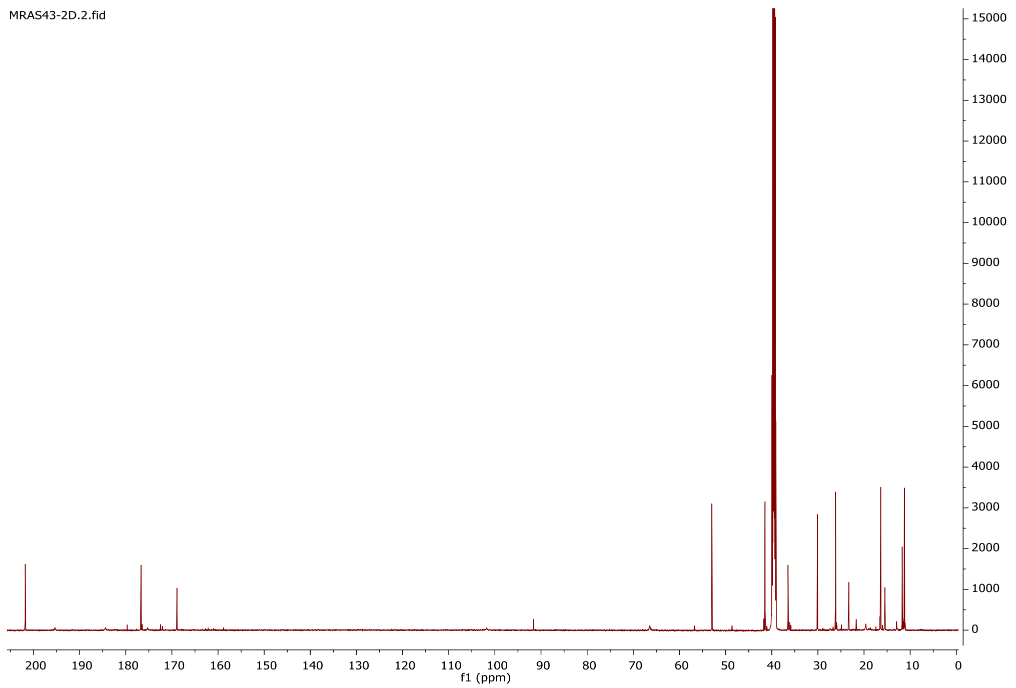

Figure S19: ^{13}C NMR spectrum of compound **8** (600 MHz, DMSO– *d*$_6$, 298 K)

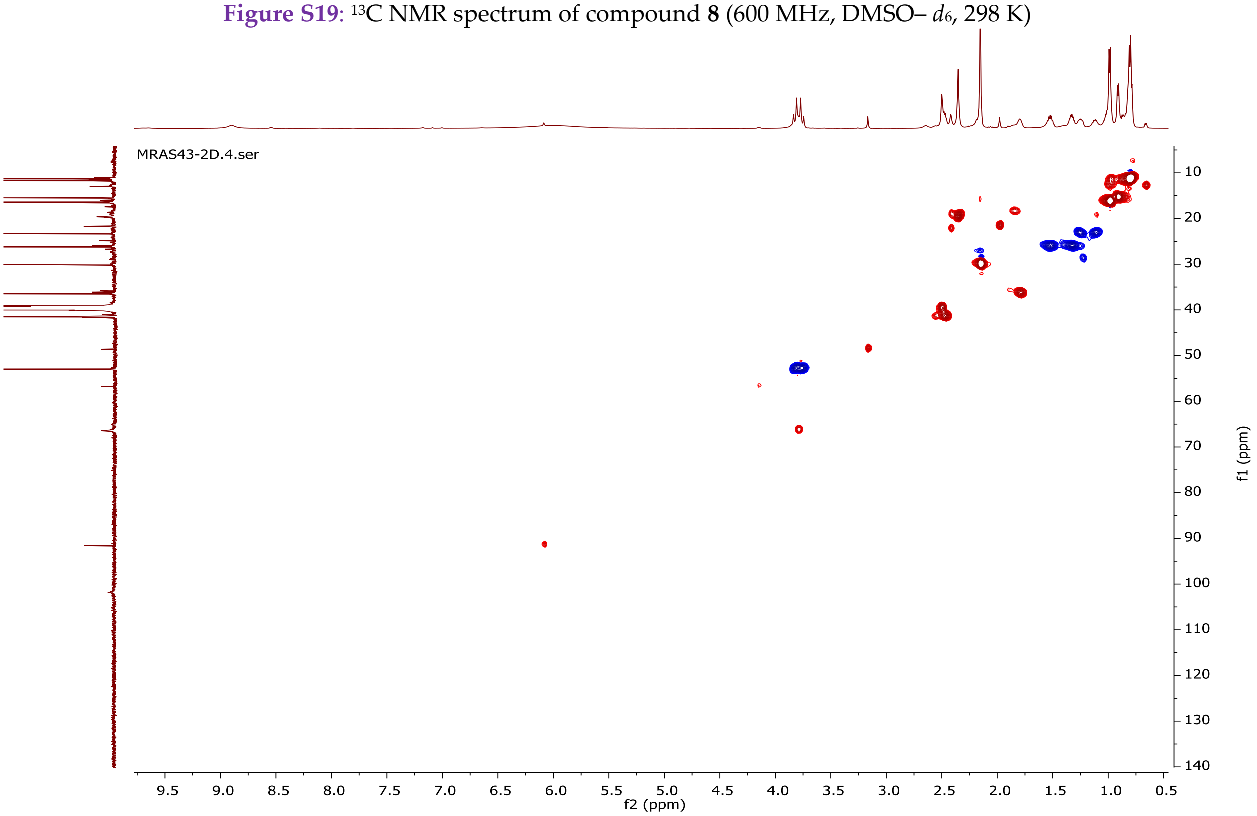

Figure S20: HSQC spectrum of compound **8** (DMSO– *d*$_6$, 298 K)

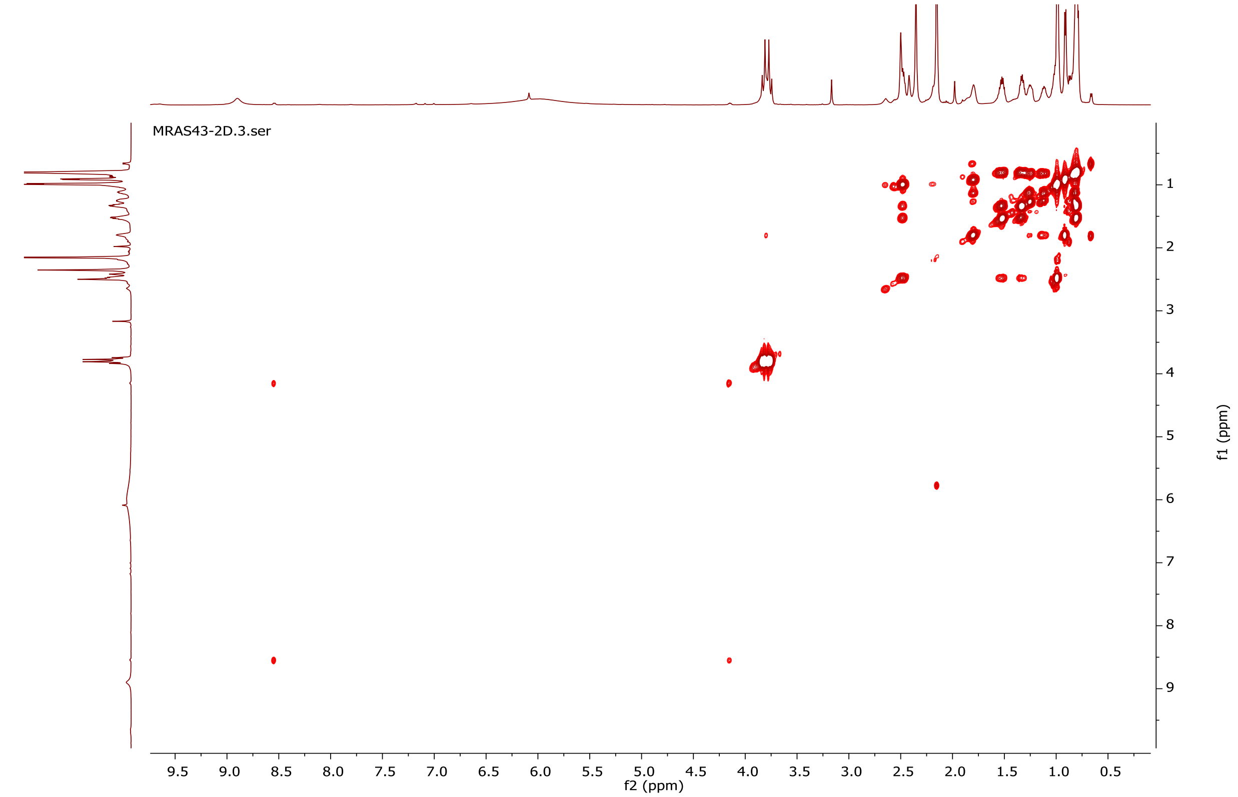

Figure S21: ^{1}H-^{1}H COSY spectrum of compound **8** (DMSO– d_6, 298 K)

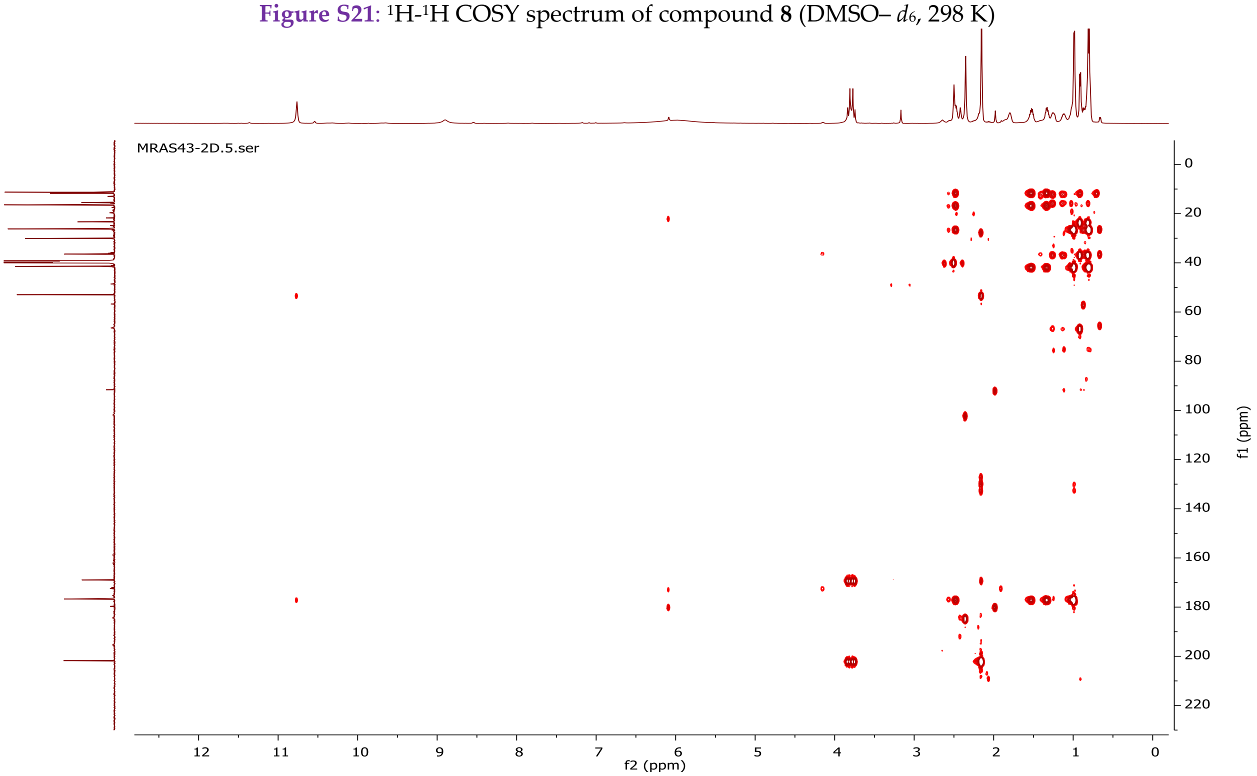

Figure S22: HMBC spectrum of compound **8** (DMSO– d_6, 298 K)

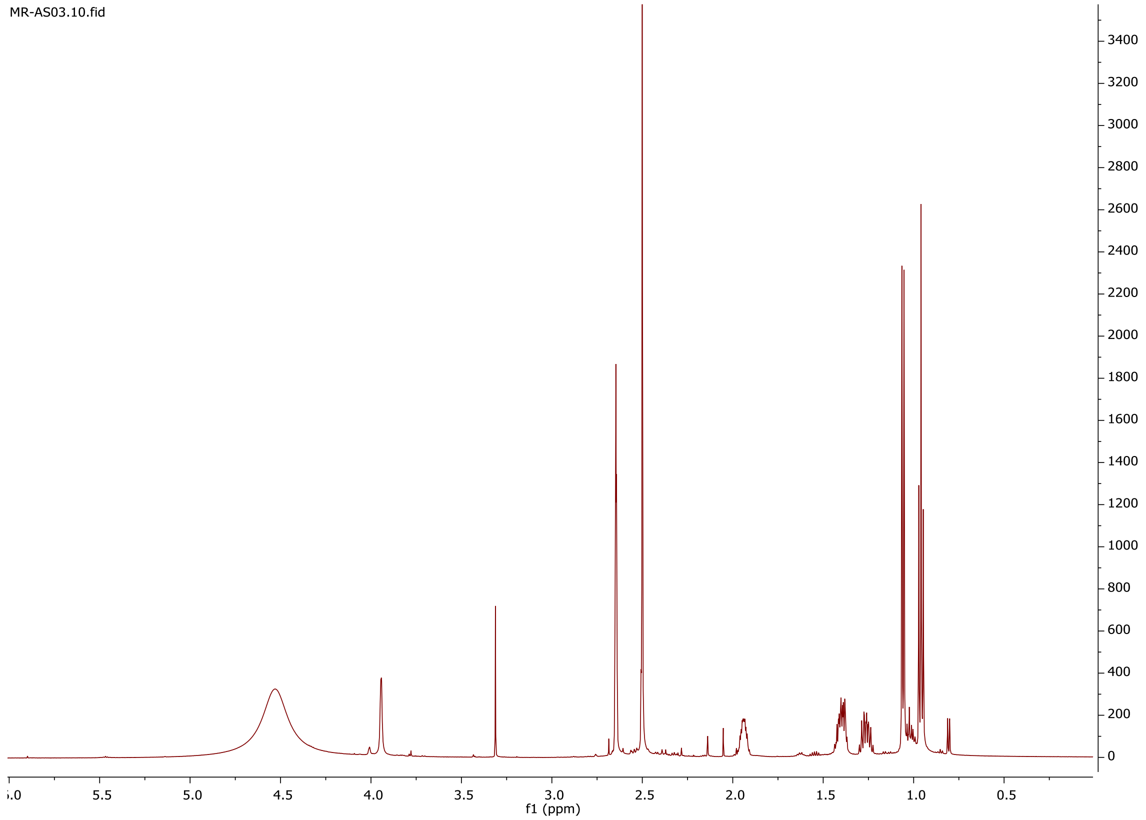

Figure S23: ^{1}H NMR spectrum of compound **9** (600 MHz, DMSO– d_6, 298 K)

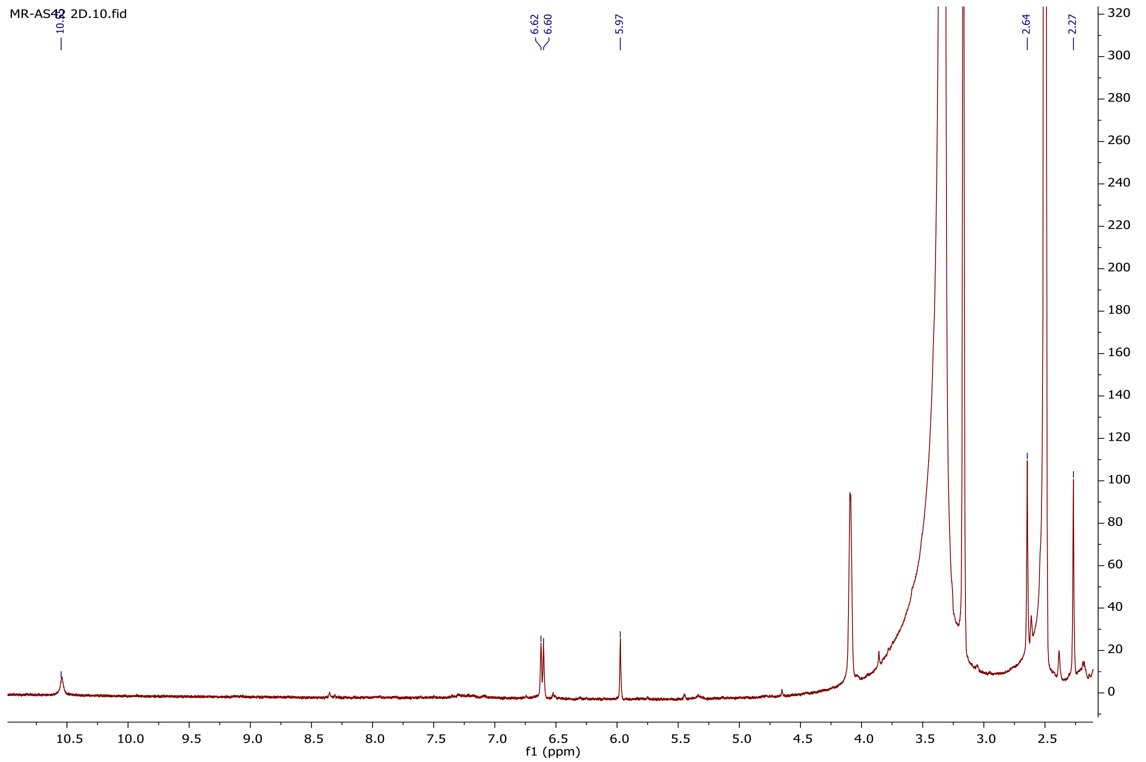

Figure S24: ^{1}H NMR spectrum of compound **10** (600 MHz, DMSO– d_6, 298 K)

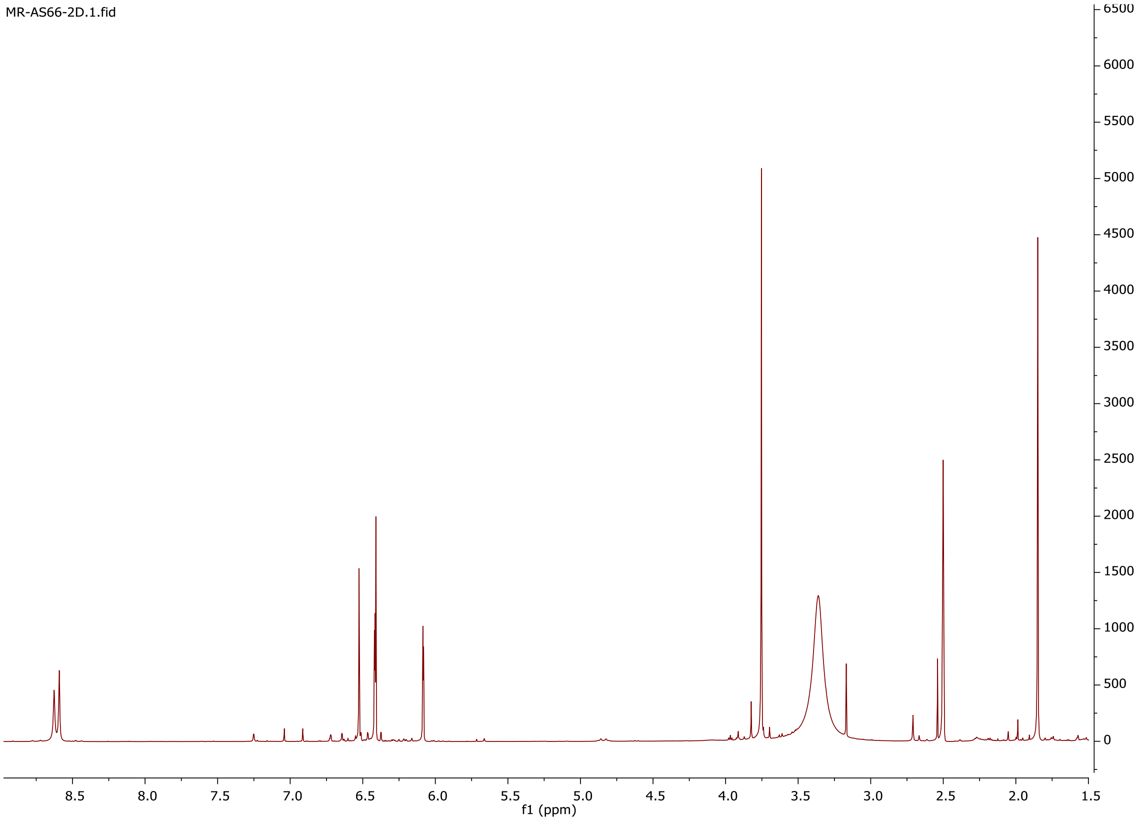

Figure S25: ^{1}H NMR spectrum of compound **11** (600 MHz, DMSO– d_6, 298 K)

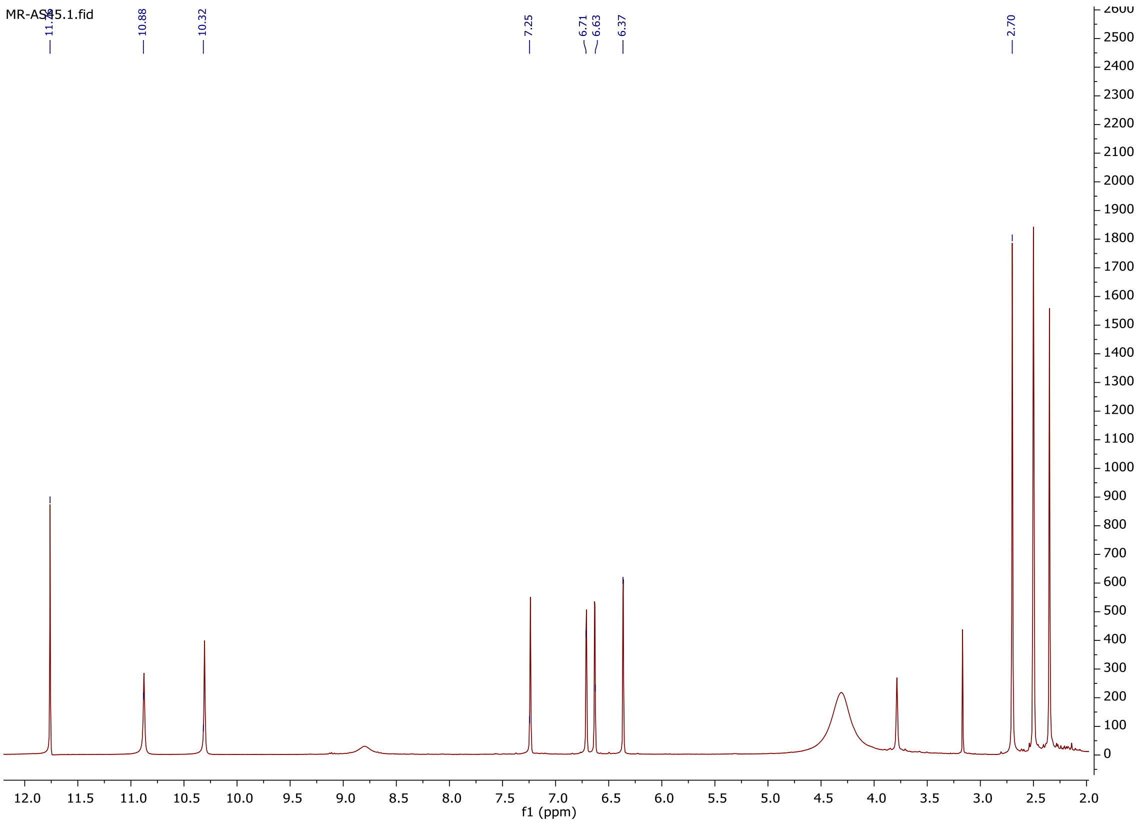

Figure S26: ^{1}H NMR spectrum of compound **12** (600 MHz, DMSO– d_6, 298 K)

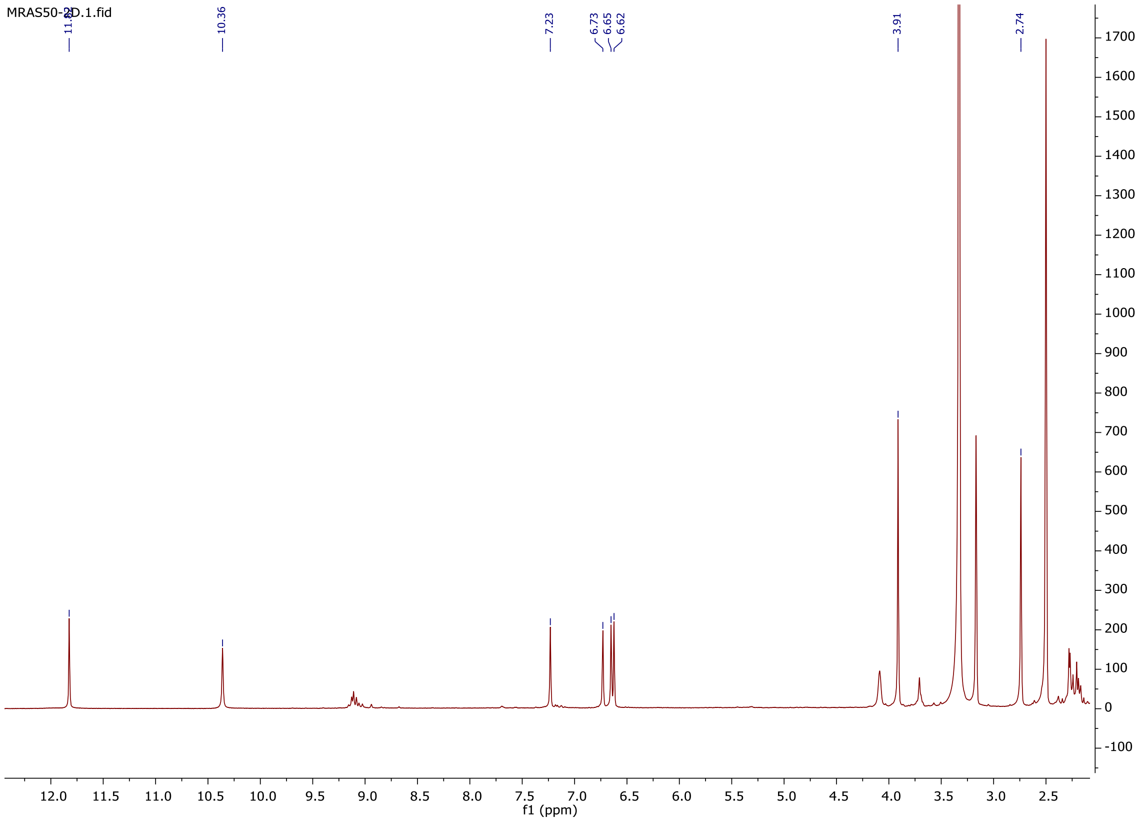

Figure S27: ^{1}H NMR spectrum of compound **13** (600 MHz, DMSO– d_6, 298 K)

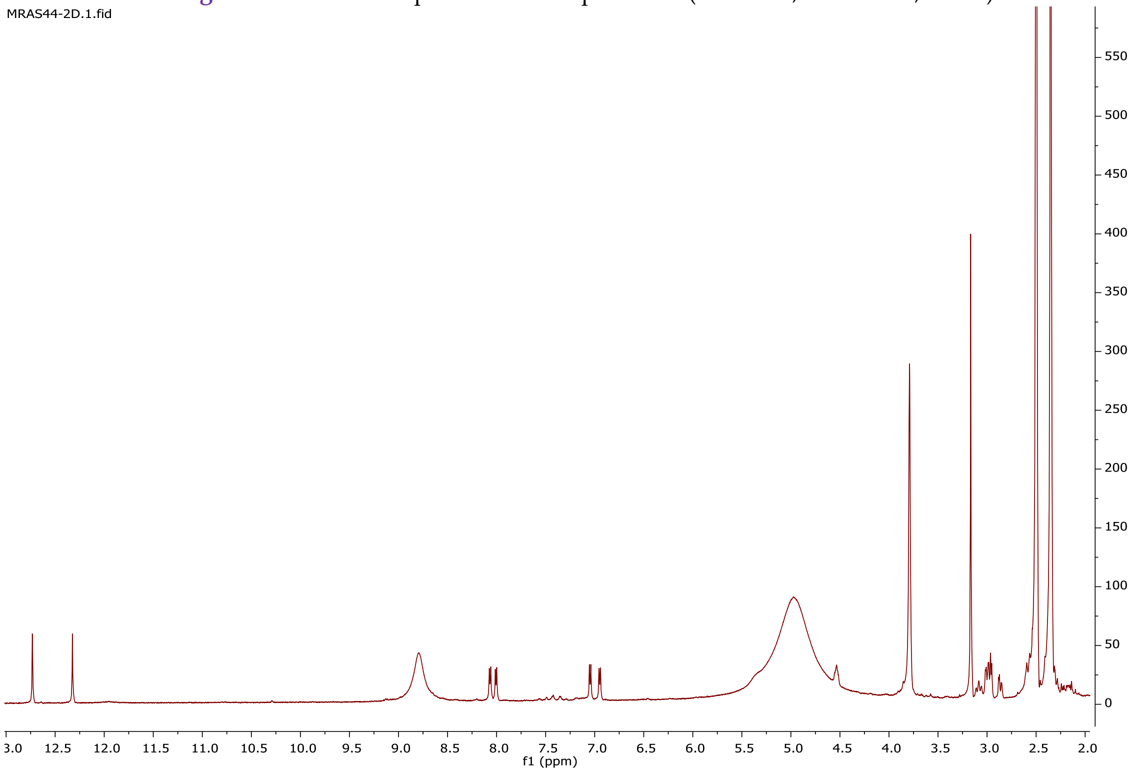

Figure S28: ^{1}H NMR spectrum of compound **14** (600 MHz, DMSO– d_6, 298 K)

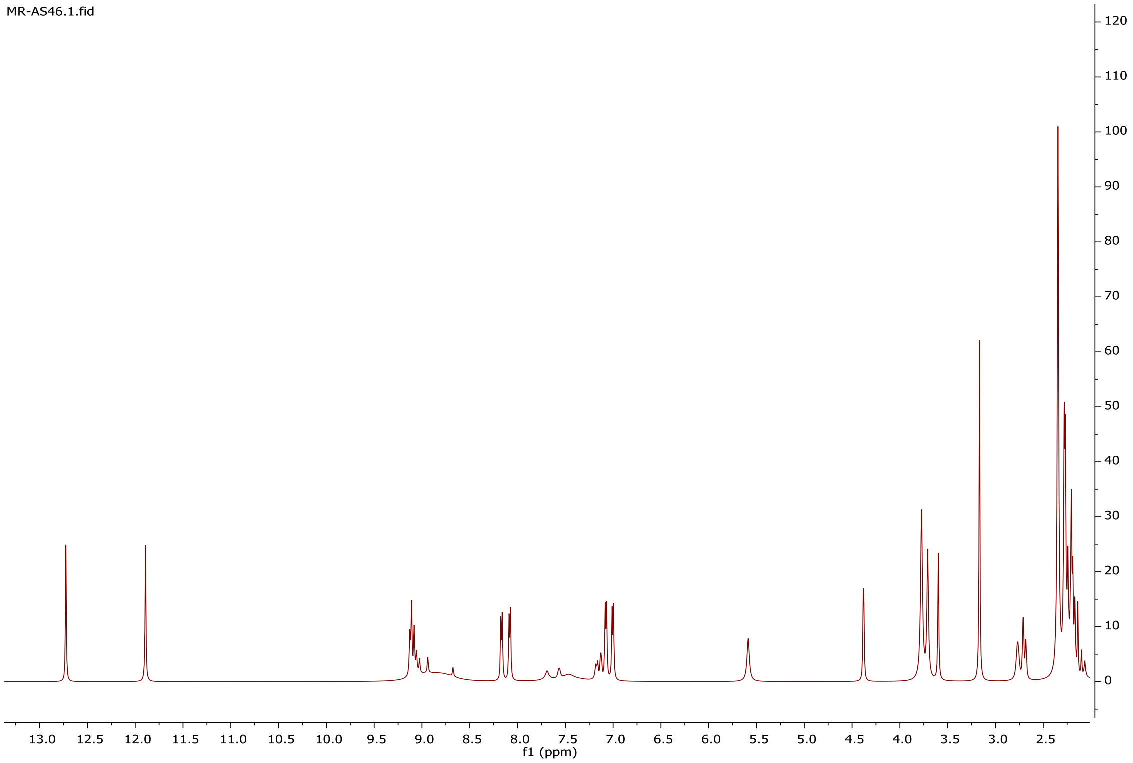

Figure S29: ^{1}H NMR spectrum of compound **15** (600 MHz, DMSO– d_6, 298 K)

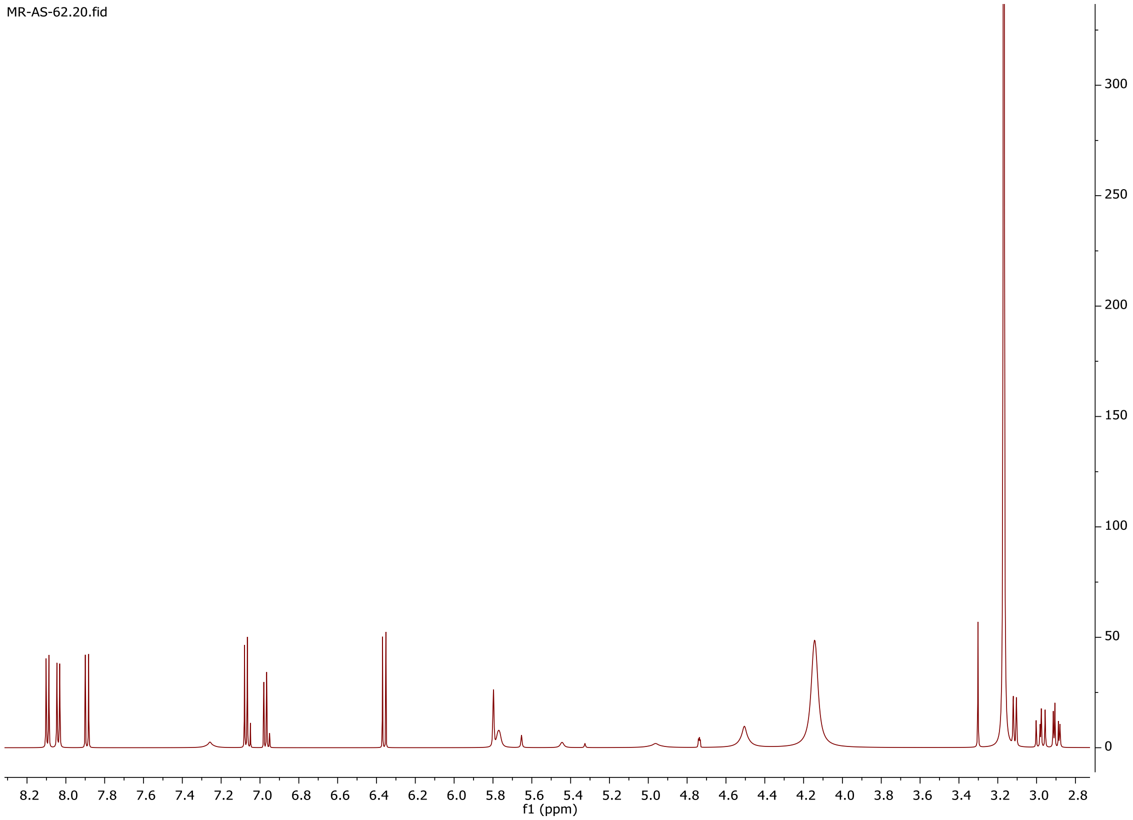

Figure S30: ^{1}H NMR spectrum of compound **16** (600 MHz, DMSO– d_6, 298 K)
